# Distinction of Neurons, Glia and Endothelial Cells in the Cerebral Cortex: An Algorithm Based on Cytological Features

**DOI:** 10.3389/fnana.2016.00107

**Published:** 2016-11-01

**Authors:** Miguel Á. García-Cabezas, Yohan J. John, Helen Barbas, Basilis Zikopoulos

**Affiliations:** ^1^Neural Systems Laboratory, Department of Health Sciences, Boston UniversityBoston, MA, USA; ^2^Human Systems Neuroscience Laboratory, Department of Health Sciences, Boston UniversityBoston, MA, USA

**Keywords:** nucleus, heterochromatin, stereology, astrocytes, microglia, oligodendrocytes, human, monkey

## Abstract

The estimation of the number or density of neurons and types of glial cells and their relative proportions in different brain areas are at the core of rigorous quantitative neuroanatomical studies. Unfortunately, the lack of detailed, updated, systematic and well-illustrated descriptions of the cytology of neurons and glial cell types, especially in the primate brain, makes such studies especially demanding, often limiting their scope and broad use. Here, following an extensive analysis of histological materials and the review of current and classical literature, we compile a list of precise morphological criteria that can facilitate and standardize identification of cells in stained sections examined under the microscope. We describe systematically and in detail the cytological features of neurons and glial cell types in the cerebral cortex of the macaque monkey and the human using semithin and thick sections stained for Nissl. We used this classical staining technique because it labels all cells in the brain in distinct ways. In addition, we corroborate key distinguishing characteristics of different cell types in sections immunolabeled for specific markers counterstained for Nissl and in ultrathin sections processed for electron microscopy. Finally, we summarize the core features that distinguish each cell type in easy-to-use tables and sketches, and structure these key features in an algorithm that can be used to systematically distinguish cellular types in the cerebral cortex. Moreover, we report high inter-observer algorithm reliability, which is a crucial test for obtaining consistent and reproducible cell counts in unbiased stereological studies. This protocol establishes a consistent framework that can be used to reliably identify and quantify cells in the cerebral cortex of primates as well as other mammalian species in health and disease.

## Introduction

Quantitative anatomic studies in the brain require precise morphological criteria to identify cells and their components in stained sections examined under the microscope. Precise criteria are crucial for obtaining consistent and reproducible cell counts in unbiased stereological studies. A practical, reliable and specific staining of all cells within distinct regions is prerequisite for estimating consistently the entire population of neurons, and specific types of glia, such as astrocytes, oligodendrocytes, and microglia. Some immunohistochemical markers label neurons and glial cells differentially and help distinguish and identify cell types for stereological counts. These approaches are widely used to identify specific neurochemically/molecularly-defined subtypes of cells, including for example the identification or quantification of distinct neurochemically-defined subtypes of inhibitory neurons (DeFelipe, 1997; Dombrowski et al., 2001; Zikopoulos and Barbas, 2013). However, these methods can only be used to quantify ratios of distinct subtypes within the entire population of neurons and glia, when complemented by Nissl, exemplified in quantitative studies of the proportion of inhibitory neurons in cortical areas (Beaulieu et al., 1992; Gabbott and Bacon, 1996; Gabbott et al., 1997). That is because markers and experimental procedures vary significantly in their sensitivity or specificity and may not label the entire population of a given cell type. For instance, glial fibrillary acidic protein (GFAP) is specific for astrocytes in the adult nervous system, but labels mostly fibrillary and reactive astrocytes while a lot of protoplasmic and non-reactive astrocytes remain unlabeled (Connor and Berkowitz, 1985; Matthias et al., 2003; Kimelberg, 2004; Lyck et al., 2008; Sofroniew and Vinters, 2010). Similarly, several markers are used to label oligodendrocytes but none labels the entire oligodendrocyte population (Ness et al., 2005). Thus, while 2′,3′-Cyclic-Nucleotide 3′-Phosphodiesterase (CNPase) has been proposed as a candidate to identify oligodendrocytes because it stains their cell bodies in the adult brain, there are no studies attesting to its specificity (Lyck et al., 2008) and some reports suggest that this marker also labels microglia (Wu et al., 2006; Yang et al., 2014). The Cluster of Differentiation 68 (CD68), which labels both resting and activated microglia (Hulette et al., 1992; Ulvestad et al., 1994; Mittelbronn et al., 2001; Wojtera et al., 2012), may be among the most specific and sensitive immunohistochemical markers. However, CD68 labels the highly ramified cytoplasm of microglia but not the nucleus, making identification of individual microglial cells difficult (Plog et al., 2014). The calcium binding protein IBA-1 is specific for microglia and labels both the ramified and the perinuclear cytoplasm (Ito et al., 1998; Torres-Platas et al., 2014), but no studies have yet assessed whether it labels the entire population of microglial cells (Boche et al., 2013). Finally, NeuN antibodies are specific for neurons (Mullen et al., 1992; Wolf et al., 1996) and are widely used in stereological counts of the brain (Lyck et al., 2006, 2009; Azevedo et al., 2009), but some reports show that this marker does not label the entire neuronal population of some brain regions (Cannon and Greenamyre, 2009; Gusel’nikova and Korzhevskiy, 2015; Sarnat, 2015; see also Hilgetag et al., 2016 and personal observations). Besides sensitivity and specificity of antibody, other factors affect markers based on immunohistochemistry. Some of these include *post-mortem* delay, prolonged fixation and tissue embedding, which affect the reproducibility and consistency of immunostaining in human brains and can bias quantification of cell populations (Lyck et al., 2008).

Compared to immunohistochemistry, the classical Nissl technique has several advantages for quantitative studies where entire populations of cells must be assessed. Such studies in normal brain tissue form the basis for comparison across cortical regions in brains that are affected in disease. First, the Nissl technique stains the entire population of neurons and glial cell types in the same section. Second, the Nissl technique stains differentially all cell types of nervous tissue allowing distinction and identification of all cells. These features make Nissl staining the most suitable technique for labeling neurons and glial cell types in stereological counts of entire nerve cell populations in the cortex. Other advantages of Nissl staining over immunohistochemistry are the low cost of this technique and the abundant available material from different species, including human, already processed for Nissl staining in neuroscience laboratories and in curated collections around the world.

Unbiased counts of neurons and glial cells in Nissl stained sections depend on the ability of the observer to discriminate cellular types according to their cytological features, a task requiring an experienced eye (O’Kusky and Colonnier, 1982; Christensen et al., 2007) that cannot be substituted by automated cell detection methods (Schmitz et al., 2014). Unfortunately, descriptions of neurons and glia in quantitative studies are usually brief and incomplete and the researcher has to dive in to the classical literature to find detailed cytological descriptions of neurons, astrocytes, oligodendrocytes and microglia (Ramón Y Cajal, 1896; Del Río-Hortega, 1919; Schlote, 1959). Only two modern studies describe in detail cell cytology in the brain of rats using semithin sections stained for toluidine blue (Ling et al., 1973; Gabbott and Stewart, 1987). Another study described briefly neuron and glial cell features in the human cerebral cortex stained for Nissl (Pelvig et al., 2008) and in another article, the same group confirmed their cytological findings with immunohistochemistry (Hou et al., 2012). Thus, there is a lack of detailed, updated, systematic and well-illustrated descriptions of the cytology of neurons and glial cell types, especially in the primate brain. Furthermore, potential discrepancy in distinguishing neurons and glial cell types between observers has not been tested.

In this article we provide detailed protocols to distinguish neurons and glial cell types in Nissl stained sections of the cerebral cortex. We first describe systematically the cytological features of neurons and glial cell types in the cerebral cortex of the macaque monkey and the human using thick and semithin sections stained for Nissl. We provide abundant examples of each cell type in the figures and corroborate key distinguishing characteristics of different cell types in sections labeled for specific markers (GFAP for astrocytes, Iba-1 for microglia, NeuN for neurons) and counterstained for Nissl, and in ultrathin sections processed for electron microscopy. Then we summarize the key features for cell type distinction in tables and we structure these key features in an algorithm that can be used to systematically distinguish cellular types in the primate cerebral cortex. Finally, we test the reliability among observers that use the algorithm to identify neurons and glial cell types. The summary tables and the algorithm can be used by researchers to identify and quantify cells in the cerebral cortex of primates as well as other mammalian species.

## Materials and Equipments

### Animal Cases

We analyzed cell features in Nissl stained thick sections of anterior cingulate areas 24, 25 and 32 and dorsolateral prefrontal area 46 of the macaque monkey (*n* = 12; cases AI, AN, AR, AS, AT, AY, BB, BD, BJ, BN, BS, and BT) and in Nissl stained semithin sections of anterior cingulate area 32 of the monkey (*n* = 6 cases BN, BS, and BT; 3 cases were a gift from Dr. Alan Peters: AM16, AM76, and AM129). We also analyzed cell features in the same areas in sections labeled for specific markers using immunohistochemistry (GFAP for astrocytes, NeuN for neurons; *n* = 2 cases BB and BD, Iba-1 for microglia; *n* = 3 cases AY, BB and BT) and counterstained for Nissl. Finally, we examined ultrathin sections of areas 25, 32, 46 and 17 in the electron microscope (*n* = 5 cases BI, BL, BN, and BU; case AM65 was a gift from Dr. Alan Peters). Data of monkey cases and the stains used are summarized in Table 1.

**Table 1:** **Data of monkey and human cases analyzed**.

	Sex	Age (years)	Nissl semithin section	Nissl thick section	GFAP	Iba-1	NeuN	EM
**Case (Monkey)**
AI	M	2	−	+	−	−	−	−
AN	−	−	−	+	−	−	−	−
AR	−	3	−	+	−	−	−	−
AS	F	2	−	+	−	−	−	−
AT	F	2	−	+	−	−	−	−
AY	F	3	−	+	−	+	−	−
BB	F	2	−	+	+	+	+	−
BD	M	2	−	+	+	−	+	−
BI	F	3	−	−	−	−	−	+
BJ	F	2	−	+	−	−	−	−
BL	M	3	−	−	−	−	−	+
BN	M	2	+	+	−	−	−	+
BS	F	3.5	+	+	−	−	−	−
BT	F	4	+	+	−	+	−	−
BU	M	4	−	−	−	−	−	+
AM16	M	5	+	−	−	−	−	−
AM65	F	33	−	−	−	−	−	+
AM76	F	6	+	−	−	−	−	−
AM129	F	7	+	−	−	−	−	−
**Case (Human)**
MD12112758	F	58	−	+	−	−	−	+
MD12112043	M	83	−	+	−	−	−	−
VA12103176	M	67	−	+	−	−	−	−
MD12120103	M	50	−	+	−	−	−	−
MD12112146	M	60	−	+	−	−	−	−
B-4786	M	36	−	−	−	−	−	+
B-4981	M	42	−	−	−	−	−	+
B-5343	F	41	−	−	−	−	−	+
B-6004	F	36	−	−	−	−	−	+

Animals were obtained from Primate Research Centers and procedures were designed to minimize animal suffering and reduce the number of animals used. Detailed protocols of the procedures were approved by the Institutional Animal Care and Use Committee at Harvard Medical School and Boston University School of Medicine in accordance with NIH guidelines (DHEW Publication no. [NIH] 80-22, revised 1996, Office of Science and Health Reports, DRR/NIH, Bethesda, MD, USA).

### Human *Post-Mortem* Brain Tissue

We analyzed sections of prefrontal cortex from neurotypical human *post-mortem* brain tissue stained for Nissl (*n* = 5 cases MD12112758, MD12112043, VA12103176, MD12120103, and MD12112146) and for the electron microscope (*n* = 5 cases MD12112758, B-4786, B-4981, B-5343, B-6004). We studied neurons and glial cell types in areas 11, 24, 25, 32 and 46 of the prefrontal cortex. Data of human cases are summarized in Table 1.

*Post-mortem* prefrontal brain tissue was obtained from the Harvard Brain Tissue Resource Center through the Autism Tissue Program, the Institute for Basic Research in Developmental Disabilities, the University of Maryland Brain and Tissue Bank, and Anatomy Gifts Registry. The study was approved by the Institutional Review Board of Boston University.

### Fixation and Tissue Processing

Animals were deeply anesthetized with a lethal dose of sodium pentobarbital (>50 mg/kg, intravenous, to effect) for transcardial perfusion. One group of animals (*n* = 6, cases AN, AR, AS, AT, BB, and BD) was perfused with saline followed by 4% paraformaldehyde in cacodylate buffer (0.1 M at pH 7.4). In one case (AI), the concentration of paraformaldehyde was 6% followed by postfixation in a solution of 6% paraformaldehyde with 10% glycerol and 2% dimethyl sulfoxide (DMSO) in PB 0.1 M at pH 7.4. A second group of animals (*n* = 8 cases AY, BI, BJ, BL, BN, BS, BT, and BU) was perfused with 4% paraformaldehyde and 0.2% glutaraldehyde in 0.1 M PB (pH 7.4). After removal from the skull all brains were photographed, cryoprotected in a series of sucrose solutions (10–30% in 0.01 M PBS) and frozen in −75°C isopentane (Fisher Scientific, Pittsburg, PA, USA) for rapid and uniform freezing (Rosene et al., 1986). Brains were cut in the coronal plane on a freezing microtome at 40 or 50 μm to produce 10 matched series. The cases offered by Dr. Alan Peters (AM16, AM65, AM76, and AM129) were perfused with 1% paraformaldehyde and 1.25% glutaraldehyde in 0.1 M cacodylate buffer (pH 7.4) and postfixed for 3–7 days in 2% paraformaldehyde and 2.5% glutaraldehyde in the same buffer; pieces of areas 32 and 17 were cut into ~2 mm thick blocks, osmicated, dehydrated in a series of ascending alcohols and embedded in araldite. Semithin sections of 1 μm were cut perpendicular to the pial surface for toluidine blue staining (Peters et al., 1991a, 2008; Nielsen and Peters, 2000).

Human brains were fixed by immersion in 10% formalin. Each brain was sliced in coronal tissue slabs of 1 cm thickness. Blocks containing the areas of interest were separated, cryoprotected, frozen and cut as described for the monkey brains. Some blocks were not cryoprotected and, after embedding in agar (6%) were cut using a vibratome (Precisionary VF-700, Precisionary Instruments Inc., Greenville, NC, USA) at 50 or 75 μm.

### Nissl Staining of Thick Sections for Optical Microscopy

Sections of the prefrontal cortex of monkey and human brains were mounted on gelatin coated slides (Gelatin Type A, G8-500, Fisher Scientific, Fair Lawn, NJ, USA), dried for 10 days and stained for Nissl using thionin blue (Thionin powder, T-409, Fisher Chemicals). Briefly, sections were defatted in a mixture of 50% ethanol (Pharmco-AAPER, Brookfield, CT, USA) and 50% chloroform (C298-1, Fisher Scientific) for 1–3 h, and then rehydrated in ethanol solution of decreasing concentration (3 steps in ethanol 100%, 2 steps in ethanol 95% and 2 steps in ethanol 70%; 2 min each step) and 2 steps of distilled water (1 min each) followed by 5 min in 0.05% thionin solution (pH 4.5). Sections were then rinsed in distilled water (2 steps, 1 min each) and differentiated and dehydrated in successive increasing concentration of ethanol solutions (2 steps in ethanol 70%, 2 steps in ethanol 95%, 3 steps in ethanol 100%). Excess thionin was washed during the first step in ethanol 95% with added 1 ml of glacial acetic acid (A6283, Sigma-Aldrich; St. Louis, MO, USA). Finally, sections were cleared in xylene (UN1307, Fischer Scientific; 2 steps of 15 and 20 min) and coverslipped with Permount (Fisher Scientific) or Entellan (107960, Merck, Darmstadt, Germany).

### Nissl Staining of Semithin Sections for Optical Microscopy

Small blocks of sections from monkey and human brains containing all the layers of the cortical areas of interest (monkey: areas 25, 32, 46 and 17; human: area 32) were cut under a dissecting microscope and processed for thin sectioning and electron microscopy. Briefly, sections were postfixed in 1% osmium tetroxide with 1.5% potassium ferrocyanide in 0.1 M PB, washed in 0.1 M PB and water, and dehydrated in an ascending series of alcohols (50–100%). While in 70% alcohol, blocs were stained for 30 min with 1% uranyl acetate (EM Sciences, Hatfield, PA, USA). Subsequently, blocks were infiltrated with propylene oxide and flat embedded in araldite at 60°C. Pieces of the araldite-embedded sections were cut and re-embedded in resin blocks. In the monkey cases we cut serial semithin sections (1 μm) with a diamond knife (Diatome USA, Hatfield, PA, USA) using an ultramicrotome (Ultracut, Leica, Vienna, Austria). We prepared 1% aqueous solution with toluidine blue powder (T3260, Sigma-Aldrich) in distilled water. The solution was filtered (Millex GV filter unit, Merck Millipore, Tullagreen, Carrigtwohill, Co. Cork, Ireland) and mixed with sodium borate (1:1; S9640, Sigma-Aldrich), filtered again and diluted with 50% with 70% ethanol. Sections floating in water were mounted on gelatin coated slides and placed on a heater plate until the water evaporated. Sections were then covered with the final toluidine blue solution for 30–60 s, rinsed with water, differentiated with 70% ethanol and rinsed with water again. Sections were coverslipped as described above.

### Immunohistochemistry for Optical Microscopy

We processed a series of sections for immunohistochemistry to label cell types with specific markers. We labeled sections with a marker specific for astrocytes (GFAP; Sofroniew and Vinters, 2010), microglia (Iba-1; Ito et al., 1998; Torres-Platas et al., 2014) and for neurons (NeuN; Wolf et al., 1996) Briefly, free-floating sections were rinsed in 0.01M PBS, incubated in 0.05 M glycine, pre-blocked for 1 h in 10% normal goat serum (NGS) and 5% bovine serum albumin (BSA) with 0.2% Triton-X and incubated overnight in primary antibody against GFAP (rabbit anti-GFAP, diluted 1:500, Sigma-Aldrich; cases BB, BD), Iba-1 (goat anti-Iba-1, diluted 1:1000, Abcam, Cambridge, MA, USA; cases BB, AY, and BT) and NeuN (mouse anti-NeuN, diluted 1:200, Chemicon International Inc., Temecula, CA, USA; cases BB, BD). Sections were rinsed in PBS and incubated for 4 h in secondary biotinylated goat anti-mouse IgG or mouse anti-rabbit IgG or horse anti-goat (1:200, in PBS, 1% NGS/Normal Horse Serum, 1% BSA, 0.1% Triton-X, Vector Laboratories, Burlingame, CA, USA), followed by 1 h in an avidin-biotin horseradish peroxidase complex (AB-HRP kit; Vectastain PK-6100 ABC Elite kit, Vector Laboratories; diluted 1:100 in 0.01M PBS with 0.1% Triton X-100). Sections were rinsed and developed for the peroxidase-catalyzed polymerization of diaminobenzidine (DAB; DAB kit, Vector Laboratories or Zymed Laboratories Inc., South San Francisco, CA, USA; 0.05% DAB, and 0.004% H_2_O_2_ in PBS) for 2–3 min under microscopic control. Sections were counterstained with thionin blue as described above for nuclear description of GFAP, Iba-1 and NeuN labeled cells.

### Ultrathin Sections for Electron Microscopy

We also cut from blocks serial ultrathin sections (50 nm) of the monkey and human cortex using an ultramicrotome (Ultracut, Leica) with a diamond knife (Diatome USA). Ultrathin sections were collected on a single slot Pioloform-coated grids and scoped in the electron microscope (100CX, JEOL, Peabody, MA, USA).

### Photography

We photographed cellular profiles from thick and semithin Nissl stained sections using an optical microscope (Olympus optical microscope, BX51) with a CCD camera (Olympus DP70) connected to a personal computer with a commercial imaging system (DP Controller). Photographs were taken with the 100× objective (Olympus UPlanFL N 100×/1.30 Oil Iris ∞/0.17/FN26.5) using oil immersion (Immersol^TM^, 518F, Zeiss, Oberkochen, Germany). In the electron microscope neurons and glial cell types were captured using a digital camera (ES1000W, Gatan, Pleasanton, CA, USA) at a magnification of 10,000–30,000×. Images were imported into Adobe Illustrator CC software (Adobe Systems Inc., San José, CA, USA) to assemble the figures. Minor adjustment of overall brightness and contrast were made but images were not retouched.

### Statistical Analysis of Algorithm Reliability

We developed an algorithm to distinguish cell types of the primate cerebral cortex according to cytological features. We assessed the usefulness of this algorithm by computing inter-experimenter reliability in identifying neurons and glial cell types. For this purpose we took stacks of photographs (100× lens) at different planes of focus through thick sections of the monkey brain stained for Nissl in representative sites of the cortical areas analyzed (*n* = 8, cases AN, AY, BJ, BS, and BT). The photographs of each site were ordered in a PowerPoint presentation (Office 365, Microsoft) and eight raters used the cytological algorithm to group cells from these sites into five cell types (neurons, astrocytes, oligodendrocytes, microglia and endothelial cells). Cells that were in focus and were to be included in the analysis were numbered sequentially, in the order they appeared in the PowerPoint presentation, whereas cells that were not in focus in the stack were excluded. Raters were required to make a decision for each cell profile and record their decisions in a data sheet (Excel, Office 365, Microsoft). Raters had variable experience in neurocytology, ranging from very experienced (*n* = 3) to less experienced (*n* = 5), and included researchers from multiple groups and labs. Two tests were administered, the first with 236 cells, and the second with 114 cells. After the first test, there was a brief session for additional explanation of the algorithm (as in “Potential Pitfalls and Ambiguous Profiles” Section). Data analysis was performed on the cumulative dataset as well as for each test individually. One of the inexperienced raters was unavailable to perform the second test.

Inter-rater agreement was estimated using Krippendorff’s alpha (Hayes and Krippendorff, 2007), and was computed using a custom script written in MATLAB (MATLAB and Statistics Toolbox Release R2015b, The MathWorks, Inc., Natick, MA, USA). Krippendorff’s alpha ranges from 0 to 1, where 0 represents chance agreement, and 1 represents perfect agreement. Determining Krippendorff’s alpha requires computing a coincidence matrix of pairwise rater categorizations. The elements of this matrix are the sum total of *pairs* of rater categorizations for each category pair. Thus the diagonal elements of the coincidence matrix are the total number of agreements between all pairs of raters. The off-diagonal elements are the total number of pairs of disagreements between all pairs of raters. For *N* cells and *m* raters, there are *N × m × (m − 1)* possible pairs of ratings. It should be noted that these pairs are permutations rather than combinations, so each combination of two cell ratings is counted twice. For example, a cell may be labeled as a neuron by rater A and as an astrocyte by rater B. This adds 1 to the neuron-astrocyte term of the matrix, as well as to the astrocyte-neuron term. In other words, the pairs {rater A, rater B} and {rater B, rater A} both contribute to the coincidence matrix. For this reason, if rater A and rater B agree that a cell is a neuron then 2 is added to the neuron-neuron term, which is along the diagonal of the coincidence matrix. If there are no disagreements, then only the diagonal terms of the coincidence matrix have non-zero values. The number of disagreements is compared with the number of possible disagreements expected if raters operated at chance levels. The ratio thus computed is subtracted from one to yield Krippendorff’s alpha. The coincidence matrix can also provide insight into the sources of disagreement among raters—the off-diagonal terms indicate which disagreements are most common. For further details on this statistical procedure, see Hayes and Krippendorff (2007).

## Stepwise Procedures

**Figure 1:**
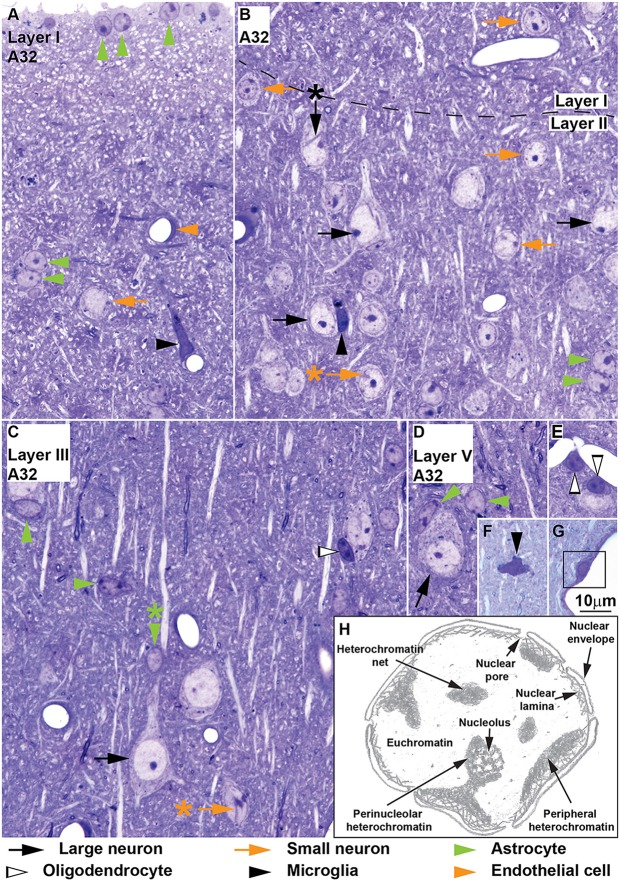
**Neurons and glial cell types in area 32 of the monkey cerebral cortex, semithin sections stained with toluidine blue and sketch of used nuclear structure terms.** *Large neurons* (black arrows) are easy to distinguish because of their large cytoplasm, large “empty” appearing nucleus and prominent nucleolus (**B–D**); large neurons may show nuclear envelope folding (**B**, black arrow with asterisk points to folding). The key to distinguishing *small neurons* (orange arrows) from astrocytes is the constant presence of a patent rim of cytoplasm encircling the nucleus in small neurons **(A–C)**, folding of the nuclear envelope (**B,C**, orange arrows with asterisk point to folding), absence of thick rim of peripheral heterochromatin and the nucleolus surrounded partially with thick clumps of heterochromatin (**B**, layer I). The cytoplasm of *astrocytes* (green arrowheads) is only visible partially with the exception of those astrocytes in layer I forming the glia limitans lining the surface of the cortex (**A**, vertical green arrowheads); other astrocytes in layer I and in other layers are found in the middle of the neuropil either in pairs (**A,B**, horizontal green arrowheads) or isolated (**C,D**, horizontal green arrowheads); some astrocytes are neuron satellite glial cells (**C**, vertical green arrowhead; **D**, diagonal green arrowhead) mostly adjacent to neuron bodies, but also to apical dendrites (**C**, vertical green arrowheads with asterisk). *Oligodendrocytes* (silhouette arrowheads) have round or ovoid nuclei that are darkly stained with 1–3 thick granules of heterochromatin located in the middle of the nucleus; oligodendrocytes may be satellites to neurons **(C)** or to blood vessels **(E)**. *Microglia* (black arrowheads) may have elongated **(A)** or polylobular **(F)** nuclei but their nucleus can also be round **(B**, compared to oligodendrocyte in **C)**; microglial cells may be satellites to blood vessels **(A)** or to neurons **(B)** or found in the neuropil **(F)**. **(G)** Pericytes (surrounded by black square) are found within the basal membrane of the endothelium. **(H)** Hand drawing of nuclear structures used in the description of Nissl-stained cells. Calibration bar in **(G)** applies to **(A–G)**.

**Figure 2:**
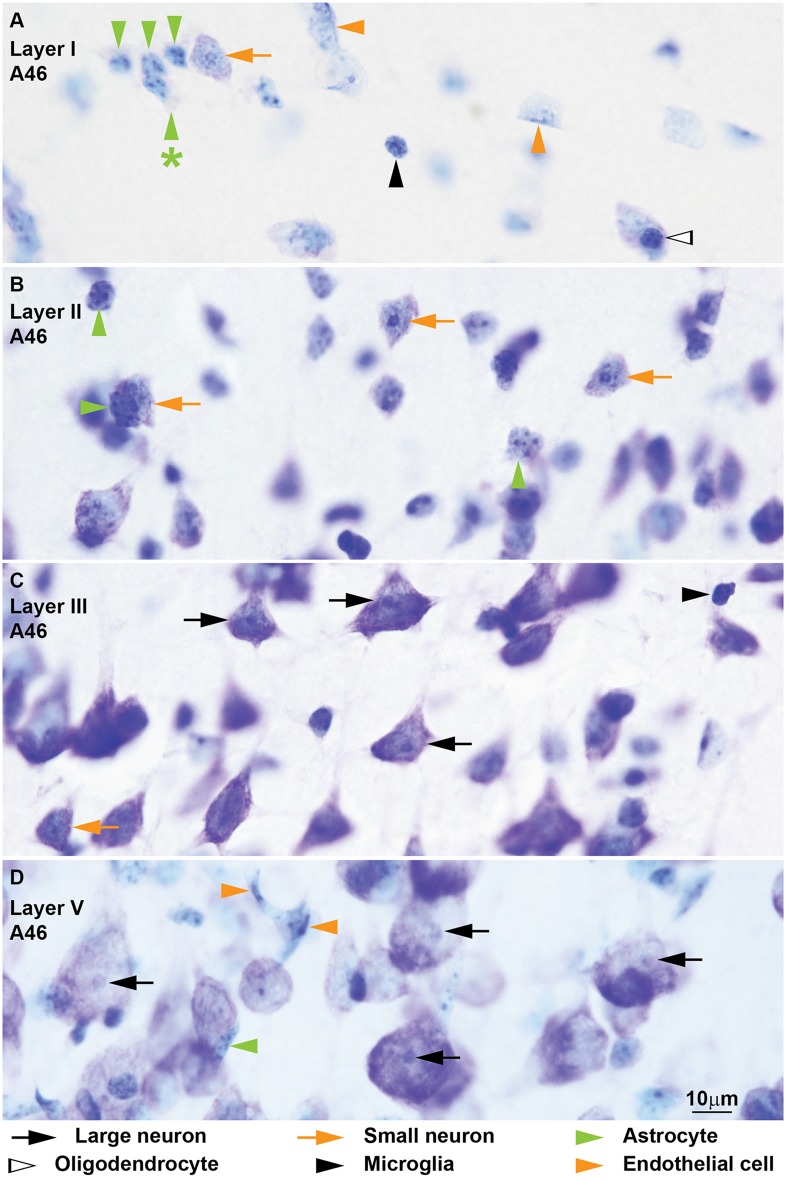
**Neurons, glial cell types and endothelial cells in coronal sections from area 46 of the monkey cerebral cortex stained with Nissl.** *Large neurons* (black arrows) are easy to identify because of their abundant cytoplasm and their large nucleus without heterochromatin granules and with distinct nucleolus **(C,D)**. *Small neurons* (orange arrows) show a rim of cytoplasm around the nucleus; the euchromatin appears light blue and contains several granules of heterochromatin, some of which form thick clumps around the nucleolus **(A–C)**. Several *astrocytes* (green arrowheads) are shown in layer I **(A)** next to a small neuron (orange arrow) for comparison; astrocytes do not show stained cytoplasm around the nucleus, but in layer I threads of rarefaction in the neuropil can be seen (**A**, green arrowhead with asterisk points to threads); they have some heterochromatin granules under the nuclear envelope and some in the middle of the nucleus not connected to peripheral heterochromatin. Some astrocytes are satellites to neurons (**B**, horizontal green arrowhead). The nuclei of *oligodendrocytes* (**A**, silhouette arrowhead) are small, round and darkly stained. The nuclei of most *microglia* (black arrowheads) are easy to distinguish from oligodendrocytes because of their irregular shape **(C)**; when microglia have round nuclei **(A)** they can be distinguished from oligodendrocytes because their heterochromatin granule area is thinner and forms a net across the nucleus **(A)**. The nuclei of *endothelial cells* (orange arrowheads) curve and mold to take the form of the wall of capillaries; endothelial nuclei have several heterochromatin granules under the nuclear envelope and in the central parts of the nucleus the euchromatin stains lightly and has a watery texture (**A,D**, see also Figure 3F). Calibration bar in **(D)** applies to **(A–D)**.

**Figure 3:**
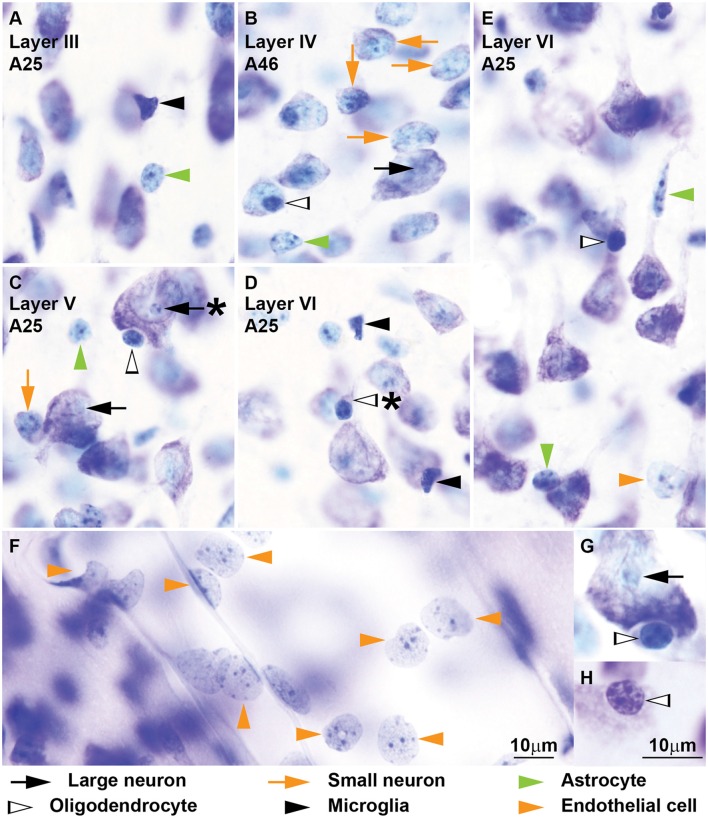
**Neurons, glial cell types and endothelial cells in coronal sections from areas 25 and 46 of the monkey cerebral cortex stained with Nissl.** Further examples of neurons and glial cell types of the monkey cerebral cortex are shown. The nucleus of *large neurons* (black arrows) appears “empty” **(B,C,G)**; some large neurons show only one or two small heterochromatin granules around the nucleus (**C**, black arrow with asterisk). *Small neurons* (orange arrows) are abundant in layer IV of area 46 **(B)**, but they are also found in other layers **(C)**; the presence of a continuous rim of cytoplasm encircling the nucleus is a useful feature to distinguish small neurons from astrocytes **(B)**. Some *astrocytes* (green arrowheads) can be satellites to neuron bodies (**E**, vertical green arrowhead) or apical dendrite satellites (**E**, horizontal green arrowhead); others are found individually in the middle of the neuropil **(A–C)**. Satellite *oligodendrocytes* (silhouette arrows) show perinuclear halo both when they are adjacent to a neuron body **(B,C,G)** or to apical dendrites **(E)**; the nuclei of oligodendrocytes have 1–4 large granules of heterochromatin of different sizes **(G,H)**; pinkish cytoplasmic crescent is a useful feature to identify oligodendrocytes (**D**, silhouette arrow with asterisk points at crescent). Typical *microglia* (black arrows) have darkly stained nuclei, generally with irregular shape **(A,D)**. Several examples of *endothelial cells* (orange arrowheads) on the wall of a medium size blood vessel are shown in **(F)**. Their nuclei have several heterochromatin granules under the nuclear envelope and in the central parts of the nucleus the euchromatin stains lightly and has a watery/rough texture, Calibration bar in **(F)** applies to **(A–F)**; calibration bar in **(H)** applies to **(G,H)**.

**Figure 4:**
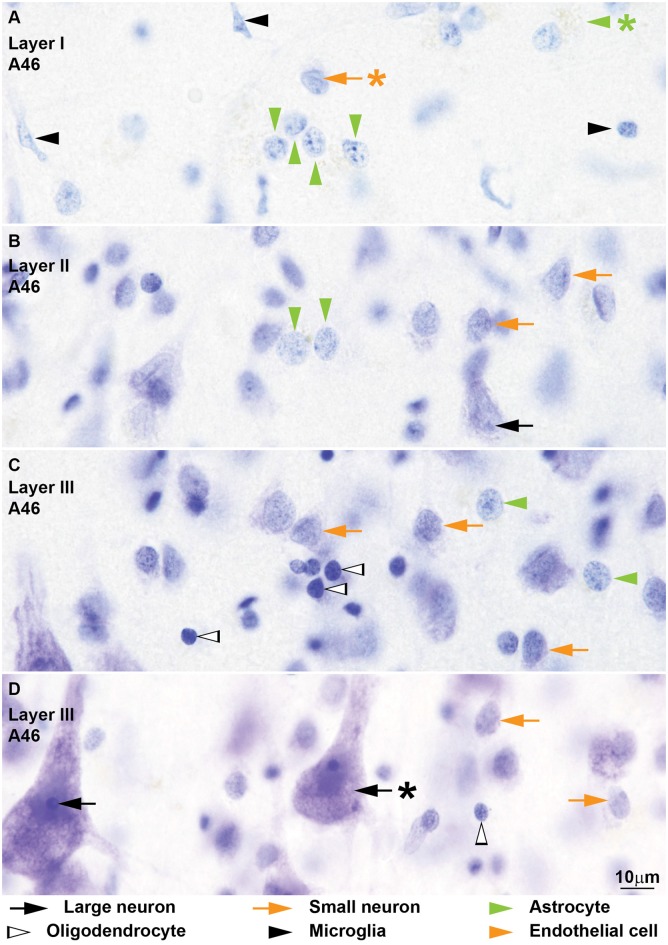
**Neurons and glial cell types from coronal sections through area 46 of the human cerebral cortex stained with Nissl.** The cytoplasm of *large neurons* (black arrows) in the human cerebral cortex usually shows lipofuscin granules (**D**, black arrow with asterisk points to lipofuscin). The nucleus is large and appears “empty” with one distinct nucleolus **(B,D)**. The cytoplasm of *small neurons* (orange arrows) is thinner and can have some lipofuscin granules **(A–D)**. In most cases the nuclear envelope shows one longitudinal folding (**A**, orange arrow with asterisk points at folding) and a small nucleolus is attached to the nuclear envelope **(A)**. Nissl stain does not color the cytoplasm of human *astrocytes* (green arrowheads) in the cortex **(A–C)** and most of these cells show a pocket of granule inclusions in the cytoplasm next to the nucleus (**A**, green arrowhead with asterisk points to inclusions); both features are useful to distinguish astrocytes from small neurons. The nuclear envelope of astrocytes is smooth without folding. Human *oligodendrocytes* (silhouette arrows) have small and darkly stained round nuclei **(C,D)**; they have more heterochromatin granules which are more evenly distributed in the nucleus and under the nuclear envelope compared to monkey oligodendrocytes **(C)**; some human oligodendrocytes show lightly stained nucleus **(D)**. The nuclei of human *microglia* (black arrowheads) can adopt different shapes, but they are more elongated compared to monkey microglia **(A)**. Calibration bar in **(D)** applies to **(A–D)**.

**Figure 5:**
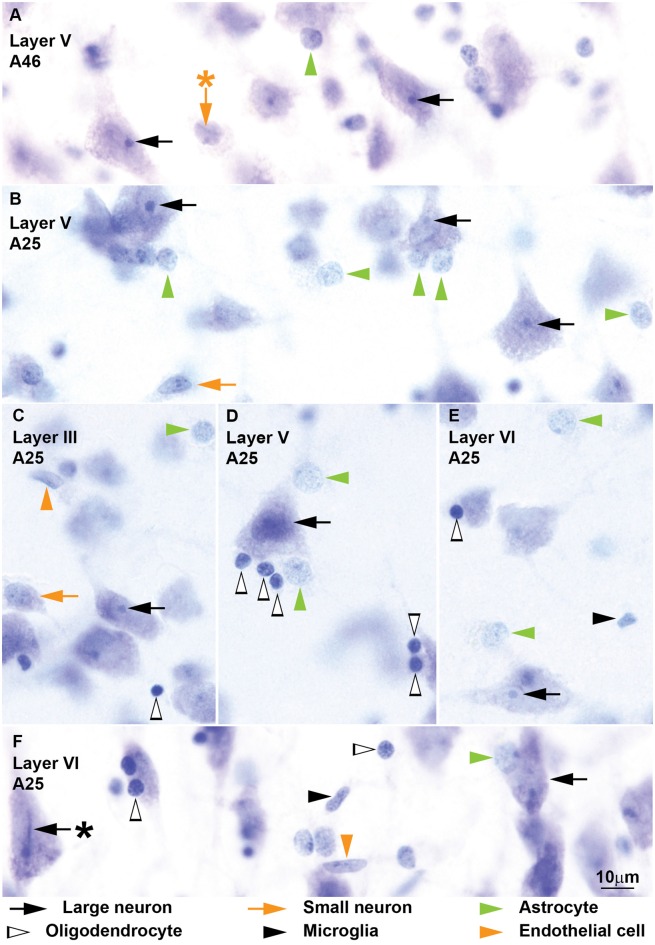
**Neurons and glial cell types from coronal sections through areas 25 and 46 of the human cerebral cortex stained with Nissl.** More examples of neurons and glial cell types of the human cerebral cortex are shown in this figure. *Large neurons* (black arrows) show lipofuscin granules in the cytoplasm and have a characteristic prominent nucleolus **(A–F)**; some large neurons show folding of the nuclear envelope (**F**, black arrow with asterisk points to folding). *Small neurons* (orange arrows) show a constant rim of cytoplasm around the nucleus **(A–C)** which may have folding of the envelope (**A**, orange arrow with asterisk points to folding). *Astrocytes* (green arrowheads) can be satellites to neurons (**A,B,D**, vertical green arrowheads) or may be found in the neuropil (**B–E**, horizontal green arrowheads). Some *oligodendrocytes* (silhouette arrowheads) are also satellites to neurons **(D,E)**; others are found in the neuropil **(C)**; most oligodendrocytes are darkly stained but a few are lighter with euchromatin that is darker than in astrocytes (**F**, horizontal silhouette arrowhead). *Microglia* (black arrowheads) nuclei may have irregular shape **(E)** or be elongated **(F)**. The nuclei of *endothelial cells* (orange arrowheads) mold to the tubular shape of capillaries **(C,F)**. Calibration bar in **(E)** applies to **(A–E)**.

**Figure 6:**
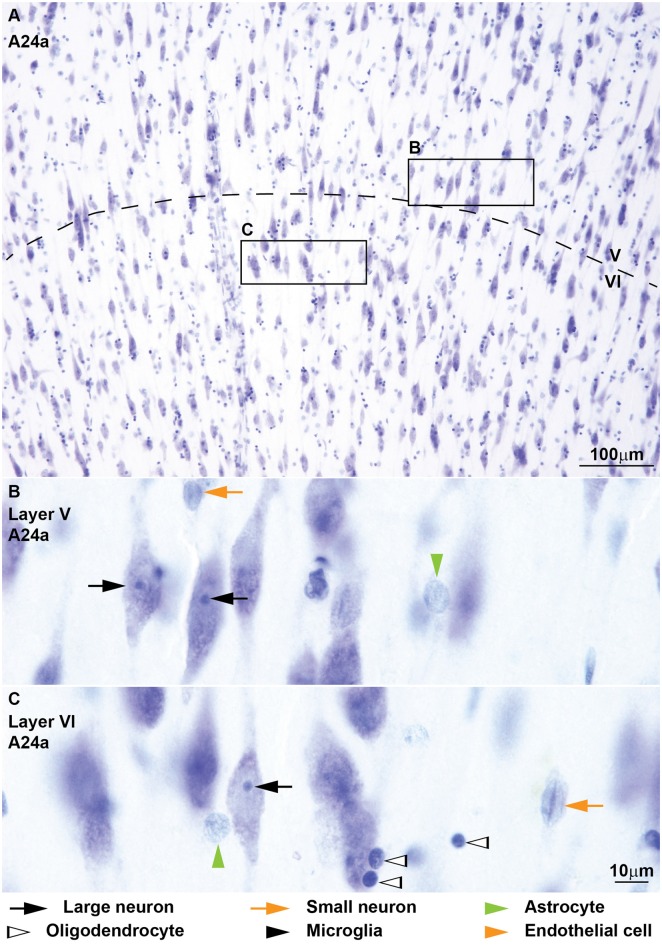
**von Economo neurons in the deep layers of subgenual area 24 of the human cerebral cortex stained with Nissl. (A)** Most large neurons in the deep layers (V-VI) of subgenual area 24 are fusiform and large pyramids are scarce. The nuclei of large fusiform neurons in layer V **(B)** and in layer VI **(C)** show a prominent nucleolus as pyramids in other areas. Calibration bar in **(C)** applies to **(B,C)**.

**Figure 7:**
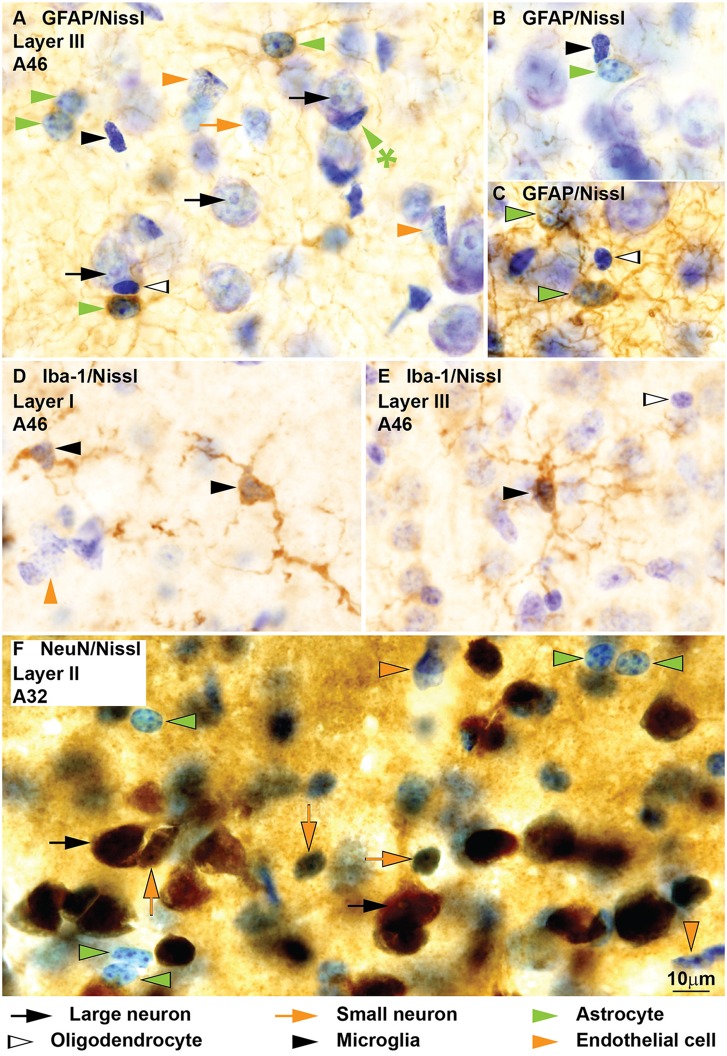
**Neurons, glial cell types and endothelial cells in coronal sections from areas 32 and 46 of the monkey cerebral cortex labeled for glial fibrillary acidic protein (GFAP), Iba-1 and NeuN and counterstained with Nissl.** GFAP labels the perinuclear cytoplasm of *astrocytes* (green arrowheads) and their processes **(A–C)**. Some astrocytes, like the satellite to a neuron in (**A**; green arrowhead with asterisk) are not labeled for GFAP. Nissl counterstaining shows the heterochromatin granules under the nuclear envelope and some in the heterochromatin net. Darkly stained *oligodendrocytes* (silhouette arrows, **A,C**) and *microglia* (black arrowheads, **A,B**) and lightly stained *endothelial cells* (orange arrowheads, **A**) are not labeled for GFAP. Iba-1 labels the branches and the perinuclear rim of *microglia* (black arrowheads, **D,E**); the nuclei of Iba-1 labeled cells may have different shapes and the heterochromatin is composed of multiple small grains. NeuN labels the nucleus and cytoplasm of *large* (black arrows, **F**) and *small neurons* (orange arrows, **F**). Nissl counterstaining shows the nuclei of *astrocytes* (green arrowheads,** F**) and *endothelial cells* (orange arrowheads, **F**) without NeuN labeling. Calibration bar in **(F)** applies to **(A–F)**.

**Figure 8:**
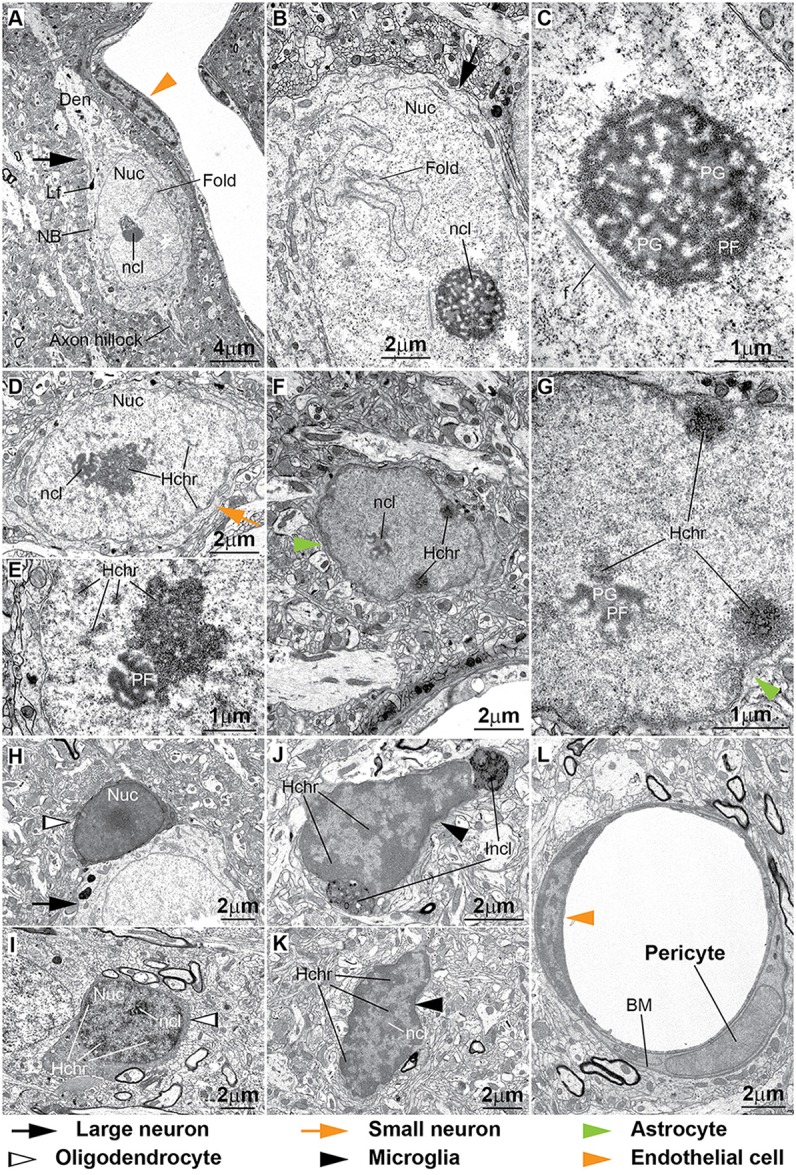
**Ultrastructural features of neurons and glial cell types in the monkey cortex. (A–E)** The cytoplasm of neurons contains Nissl bodies (NB) and lipofuscin or other lipid bodies (Lf). *Large neurons* have large lightly-stained nuclei (Nuc) with indentations or folding of the nuclear envelope (Fold in **A,B**) and a prominent nucleolus (ncl). Darkly-stained heterochromatin clumps and granules (**D**, Hchr) are more frequently seen in *small neurons* either under the nuclear envelope, the central parts of the nucleus or around the nucleolus **(D,E)**. The nucleolus of neurons is made of lobules that contain predominantly dark filaments (*pars fibrosa*, PF) or slightly lighter granules (*pars granulosa*, PG; **C,E**). In some cases fibrils (f) can be seen near the nucleolus **(C)**. **(F,G)** The envelope of the nuclei of *astrocytes* is slightly irregular and has a rim of heterochromatin under it. Clumps of heterochromatin are seen under the nuclear envelope and around the nucleolus that is composed of PF and PG. **(H,I)** *Oligodendrocytes* have dense cytoplasm and dense round nucleus in regularly fixed tissue **(H)**. In strongly fixed tissue the nuclei of oligodendrocytes show the distribution of heterochromatin and the presence of a nucleolus **(I)**. **(J,K)** The cytoplasm of *microglia* is scant and can have inclusion bodies (Incl) indenting the nuclear envelope. The shape of microglial nuclei is irregular and with its characteristic peripheral heterochromatin and heterochromatin net made of multiple fused granules. The nucleolus (ncl) is surrounded by heterochromatin. **(L)** The nuclei of *endothelial cells* have extensive peripheral heterochromatin clumps connected with the heterochromatin net. In contrast, the chromatin of *pericytes* is homogeneous. Pericytes are ensheathed by the endothelial basal membrane (BM).

**Figure 9:**
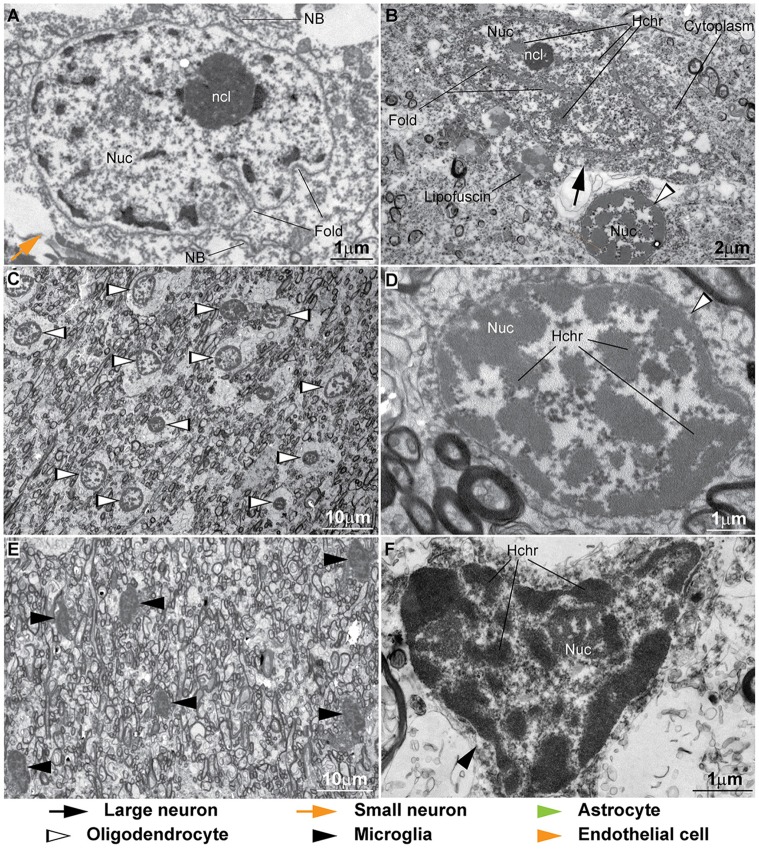
**Ultrastructural features of neurons and glial cell types in the human cortex. (A,B)** Nissl bodies (NB) and membranes of organelles in the cytoplasm of neurons often break due to poor fixation and long *post-mortem* intervals and the cytoplasm appears granulated. Heterochromatin clumps (Hchr) are seen in the different nuclear compartments. The nucleolus (ncl) is homogeneous and pars fibrosa (PF) and pars granulosa (PG) cannot be differentiated. Folds in the nuclear are seen **(B)**. The cytoplasm of human oligodendrocytes is usually unstained producing a “fried egg” appearance as shown in this neuron satellite oligodendrocyte **(B)**. **(C)** Abundant oligodendrocytes (silhouette arrowheads) can be found in the superficial white matter showing their characteristic “fried egg” appearance among myelinated axons. **(D)** Heterochromatin in human oligodendrocytes is dispersed in multiple clumps. **(E)** Some regions of the human cortex and superficial white matter show higher density of microglial cells. **(F)** The nucleus of microglial cells in human *post-mortem* tissue can be irregular in shape and shows many heterochromatin granules forming the peripheral heterochromatin and the heterochromatin net.

**Figure 10:**
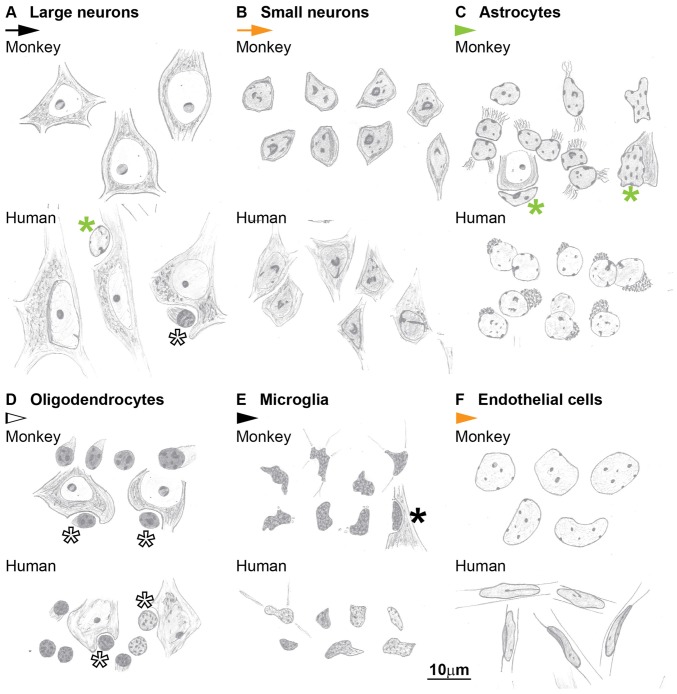
**Hand-drawings of typical examples of neuron and glial cell types from Nissl stained sections of the cerebral cortex of the monkey (upper panels) and human (lower panels).** **(A)** Large neurons, **(B)** Small neurons, **(C)** Astrocytes, **(D)** Oligodendrocytes, **(E)** Microglia, **(F)** Endothelial cells. Asterisks mark glial satellites to neurons. Calibration bar in **(F)** applies to **(A–F)**.

**Figure 11:**
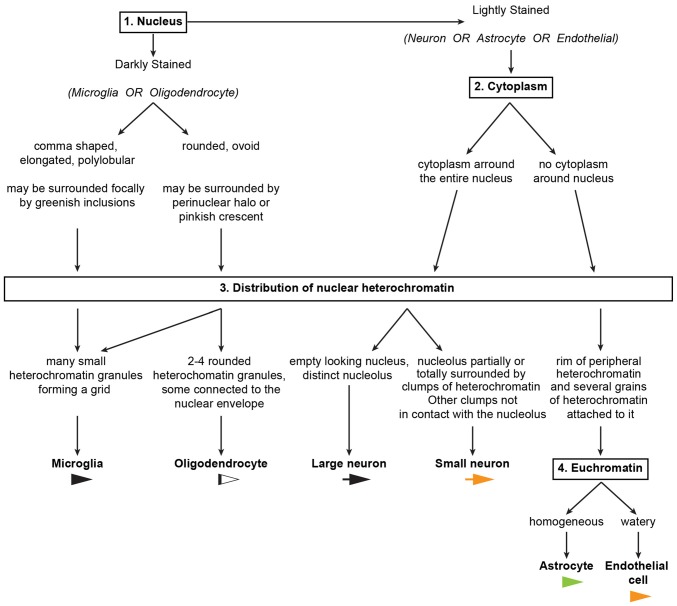
**An algorithm to systematically distinguish cell types in the primate cerebral cortex.** The key features used to distinguish neurons and glial cell types in the algorithm are summarized in Tables 2, 3. A sample Neurocytology Test and Training Set, based on a simplified version of the tests we used in this study, can be found at the Human Systems Neuroscience Lab website (http://sites.bu.edu/brainlab/neurocytology-test/), and interested researchers can take the test, record their classifications and compare their results with others.

**Figure 12:**
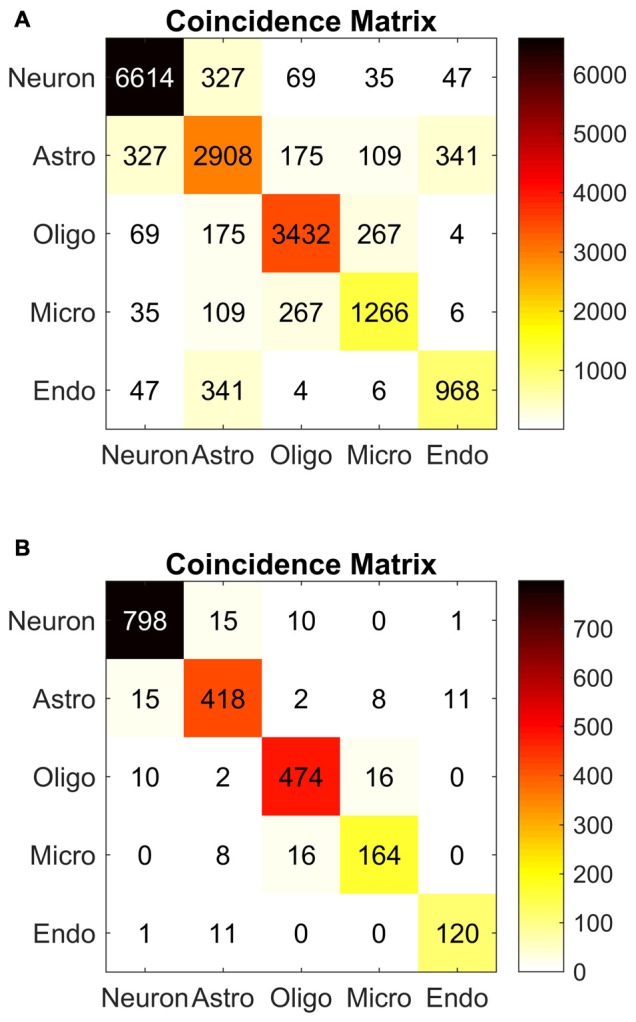
**Algorithm reliability.** Coincidence matrices used to evaluate Krippendorff’s alpha, a measure of inter-experimenter reliability. **(A)** Coincidence matrix for all raters. The corresponding alpha value was 0.79. **(B)** Coincidence matrix for experienced raters. The corresponding alpha value was 0.92. Each matrix tabulates coincidences between pairs of experimenters labeling cell types. The boxes along the diagonal represent the total number of agreements between every pair of experimenters, for the entire set of cell images. The off-diagonal numbers represent disagreements between pairs of experimenters (e. g., 327 neuron-astrocyte disagreements in the **A** matrix). Perfect agreement among experimenters would result in no off-diagonal numbers, and a Krippendorff’s alpha value of 1. Chance agreement would lead to an alpha value of 0. In both matrices, the off-diagonal numbers in the coincidence matrix were smaller than the numbers in the diagonal.

The procedures and results are organized as follows: we first describe systematically and in detail the cytological features of neurons and glial cell types in the cerebral cortex of the macaque monkey and the human using semithin and thick sections stained for Nissl (Figures 1–6). We corroborate key distinguishing characteristics of different cell types in sections processed for immunohistochemistry markers counterstained with Nissl (Figure 7) and in ultrathin sections processed for electron microscopy (Figures 8, 9). Then we summarize the core features that distinguish each cell type in easy-to-use tables (Tables 2, 3) and sketches (Figure 10) and we structure these key features in an algorithm (Figure 11) that can be used to systematically distinguish cellular types in the cerebral cortex. Finally, we test the reliability between observers that use the algorithm to identify neurons and glial cell types (Figure 12). A sample Neurocytology Test and Training Set, based on a simplified version of the tests we used in this study, can be found at the Human Systems Neuroscience Lab website^1^, where interested researchers can take the test, record their classifications and compare their results with others.

**Table 2:** **Cytological features of neurons and glial cell types in Nissl stained (toluidine blue) semithin sections of the monkey cerebral cortex**.

	Large neuron	Small neuron	Astrocyte	Oligodendrocyte	Microglia
Cytoplasm	thick rim around the nucleus	thin rim around the nucleus	scant and light	dark homogeneous perinuclear crescent	not visible
			it may have granule inclusions		it may have granule inclusions

Nuclear shape	round to ovoid	round to ovoid	ovoid with shallow concavities	round	round, elongated, comma shaped, polylobular

Nuclear envelope	sometimes folding	sometimes folding	smooth	smooth	smooth

Peripheral heterochromatin	not visible	0–2 granules	thick rim	thin rim	many small granules
			1–2 granules	0–1 granules

Heterochromatin net	not visible	1–2 granules	0–2 granules	1–2 granules	many small granules form a net
		variable size	same size	one larger than the others	same size

Perinucleolar heterochromatin	0–2 small and thin granules	1–3 thick granules	1–2 granules	1 thick granule	not visible

Nucleolus	one/two large size	one medium size	sometimes visible	sometimes visible	not visible

Euchromatin	light	light	light	dark	dark

**Table 3:** **Cytological features of neurons and glial cell types in Nissl stained (thionin) thick sections of the monkey and human cerebral cortex**.

	Large neuron	Small neuron	Astrocyte	Oligodendrocyte	Microglia
Cytoplasm	thick rim around the nucleus, heterogeneous staining	thin rim around the nucleus, heterogeneous staining	usually not visible	usually not visible	thread-like rarefaction of the neuropil
	*almost always yellow inclusions*	*often yellow inclusions*	often yellow inclusions	perinuclear halo in satellites to neurons	irregular perinuclear greenish inclusions
			*almost always yellow inclusions*
	*light and homogeneous staining*	*light and homogeneous staining*	threads in layer I	perinuclear pinkish crescent	

Nuclear shape	round to ovoid	round to ovoid	ovoid with shallow concavities	round	rounded, elongated, comma shaped, polylobular

Nuclear envelope	smooth *sometimes folding*	smooth *sometimes folding*	smooth, irregular in satellites	smooth	rarely indentations

Peripheral heterochromatin	not visible	1–2 granules	thin rim	thin rim	many small granules
			1–4 granules	1–3 granules

Heterochromatin net	not visible	1–2 granules	several granules not connected to peripheral heterochromatin	2–4 granules not connected to peripheral heterochromatin	many small granules form a net
		variable size	same size	one granule larger than others *same size*	same size

Perinucleolar heterochromatin	0–4 thin granules	2–4 thick granules	1–2 granules	1 thick granule	1–2 granules

Nucleolus	one/two large size	one medium size	sometimes visible	sometimes visible	sometimes visible
			*usually visible*

Euchromatin	not stained	light blue	light blue	dark blue	dark blue

*Specific findings for human cells are highlighted in italics*.

### Glossary for Nuclear Structures

We describe systematically the cytological features of neurons and glial cell types in the rhesus monkey and human cerebral cortex using a standardized nomenclature for nuclear structures according to Fawcett (1966) and Frost (1986), as illustrated in Figure 1H. *Nuclear membrane* is a historical term derived from light microscopy descriptions, comprising the nuclear envelope, the nuclear lamina, and the peripheral heterochromatin. *Nuclear envelope* is the membrane cisterna surrounding the entire nucleus during the interphase of the cell cycle; in our descriptions we consistently used the nuclear envelope term. *Chromatin* is the DNA-protein complex found in the interphase nucleus. *Euchromatin* is the non-condensed, open, or transcriptional chromatin. *Heterochromatin* is the condensed, closed, or non-transcriptional chromatin. Heterochromatin is divided into three compartments; *peripheral heterochromatin* is attached to the nuclear lamina under the nuclear envelope; *heterochromatin net* describes a network of heterochromatin extending from the periphery into the central portion of the nucleus; *perinucleolar heterochromatin* is found around the nucleolus which can be connected in some cases to the heterochromatin net, the peripheral heterochromatin or both. Finally, the *nucleolus* is the nuclear organelle where ribosomes are produced.

Nissl technique stains nerve cells with basic dyes like toluidine blue and thionin (Merchán et al., 2016). These colorants stain basophilic components of the cell and allow distinguishing the compartments of the nucleus. Nissl technique stains chromatin in blue; euchromatin is lightly stained while heterochromatin is darkly stained. The nucleolus, due to its protein content, is stained purple allowing distinction from clumps of dark blue stained heterochromatin (Ramón Y Cajal, 1910, 1933; Frost, 1986).

### Cytological Features of Neurons and Glial Cell Types in the Cerebral Cortex

We identified and distinguished neurons and glial cells in semithin sections of the rhesus monkey cerebral cortex stained with toluidine blue (area 32; Figure 1) and in thick sections of the rhesus monkey (areas 24, 25, 32, and 46; Figures 2, 3) and of the human cerebral cortex stained with thionin (areas 11, 24, 25, 32 and 46; Figures 4–6). What follows is a description of the most useful features to distinguish each cell type. Relevant differences in the two primate species are mentioned. Descriptions are systematic starting with the cytoplasm and proceeding to the nucleus and its components from outside in. We provide several examples of each cell type in the figures and use the same markers to point to each cell type as shown below each figure.

#### Neurons

Neurons always have visible cytoplasm around the nucleus. A small rim of cytoplasm circling the entire nucleus is a useful feature to distinguish small neurons from astrocytes. The cytoplasm of large pyramidal neurons of elderly people usually contains yellowish lipofuscin granules and smaller neurons in the human cortex also have some lipofuscin granules. In the human limbic cortices studied (areas 24, 25, 32), most large neurons were pyramidal but some large neurons in layers V and VI had fusiform shape (Figure 6) and could correspond to von Economo neurons (Watson et al., 2006). The nucleus of neurons is round or ovoid. The nuclear envelope frequently has indentations and folding, which are not usually visible in astrocytes at the light microscope. Large neurons (black arrows, Figures 1–6) virtually lack heterochromatin with the exception of 1–4 small granules attached to their large well-defined nucleolus. Small neurons (orange arrows, Figures 1–6) show some heterochromatin granules in the heterochromatin net; more heterochromatin clumps partially or totally surround the nucleolus of small neurons. The nucleolus stains purple and in large neurons is large and distinct, while in small neurons it is smaller and can be hidden by thick perinucleolar heterochromatin clumps. Some large pyramidal neurons may have two nucleoli. In the human cortex, the nucleolus of most small neurons is typically attached to a thick clump of peripheral heterochromatin. Euchromatin in small neurons is stained light blue, while in large neurons it is unstained giving the nucleus an “empty” appearance.

#### Astrocytes

The cytoplasm of astrocytes (green arrowheads, Figures 1–6), is not stained in Nissl sections, although sometimes, mostly in layer I, threads can be seen fanning out from one side of the nucleus into the neuropil. Some astrocytes have cytoplasmic yellow granular inclusions in a “pocket” next to the nucleus. In the cerebral cortex of elderly people almost every astrocyte contains inclusions. Granular inclusions are less common in neuron satellite astrocytes. The nucleus of astrocytes is ovoid with shallow concavities, like a potato, and the nuclear envelope is smooth, although perivascular and neuron satellite astrocytes may have slightly irregular nuclei with indentations. In layer I, the nuclei of astrocytes are smaller than in other layers. Astrocytes show a rim of peripheral heterochromatin under the nuclear envelope and several heterochromatin granules are attached to this rim; some granules are also found in the heterochromatin net. The nucleolus can be seen in some cases as a small purple sphere attached to one or two heterochromatin granules slightly larger than the other granules, usually located in the heterochromatin net of monkey astrocytes and under the nuclear envelope in human astrocytes. Euchromatin stains light blue. Astrocytes can be satellites to a neuron body or a neuron apical dendrite. They can also be satellites of blood vessels or found in the middle of the neuropil, often in groups of two to four.

#### Oligodendrocytes

Some oligodendrocytes (silhouette arrowheads, Figures 1–6), show a pinkish fibrillary crescent of stained cytoplasm around their nuclei. Neuron satellite oligodendrocytes typically show perinuclear halo. The nucleus of oligodendrocytes is round. The nuclear envelope is smooth and there are one or more small granules in the peripheral heterochromatin. Two to four round granules of heterochromatin, one larger than the others, form the heterochromatin net. Human oligodendrocytes have more uniformly sized and distributed heterochromatin granules within the nucleus compared to monkey oligodendrocytes. The nucleolus of oligodendrocytes, when visible, is purple and attached to one central heterochromatin granule. As a rule, euchromatin of oligodendrocytes is darkly stained but in the human cortex the nuclei of some oligodendrocytes are lightly stained. These “light” oligodendrocytes are darker than astrocytes. Oligodendrocytes can be satellites to neuron bodies, neuron apical dendrites and blood vessels. In the deep layers of the cerebral cortex, close to the white matter, they are often arranged in rows.

#### Microglia

The cytoplasm of microglial cells (black arrowheads, Figures 1–6) is not stained with toluidine blue or Nissl, although sometimes these cells show thread-like protrusions of the neuropil emerging from the pointy angles of the nucleus. Some microglial cells have small greenish inclusions in the cytoplasm in pouches close to the nucleus. Microglial nuclei can be round, ovoid, elongated, comma shaped or polylobular and may have indentations in the nuclear envelope. Microglial cells with round nuclei can be easily confused with oligodendrocytes, because both have darkly stained nuclei of the same size and shape and their cytoplasm is usually unstained. In the rhesus monkey, the key feature for distinguishing microglia with round nuclei and oligodendrocytes is the distribution of heterochromatin. The nuclei of oligodendrocytes have one or more small granules of peripheral heterochromatin and two to four rounded granules in the heterochromatin net that are darker than the euchromatin. In contrast, the nuclei of microglial cells show many peripheral heterochromatin and heterochromatin net granules of smaller size that are close to each other and form a coarse grid that obscures the euchromatin. The nucleolus of microglia is visible only in lightly stained sections. In the human cortex, the nuclei of microglial cells are more elongated and they are not as darkly stained as in the monkey, but still show multiple small granules of heterochromatin forming a grid across the nucleus. Microglial cells can be neuron and vascular satellites and also satellites to other glia. They are also found individually in the neuropil.

#### Endothelial Cells

Endothelial cells (orange arrowheads, Figures 1–6) are not glial cells but must be taken into account in stereological studies of the cortex because of their resemblance to astrocytes in the monkey and to microglia in the human. The cytoplasm of endothelial cells is not stained. The shape of endothelial nucleus is rectangular with rounded corners. Endothelial nuclei characteristically mold to the tubular shape of capillaries. The heterochromatin is composed of two to six round granules, some under the nuclear envelope and some in the heterochromatin net. Net granules are larger than peripheral granules. The nucleolus, like in astrocytes, can be seen in some cases as a small purple sphere attached to one of the heterochromatin net granules. The key feature to distinguish endothelial cells from astrocytes in the monkey cortex is the watery texture of euchromatin staining in endothelial cells. In contrast, in astrocytes euchromatin is homogeneously stained with Nissl. In the human cortex, the nuclei of endothelial cells are more elongated compared to those of the monkey and heterochromatin granules are not as sharp. The key feature to distinguish endothelial cells from microglia in the human cortex is the typical heterochromatin net of microglial cells that endothelial cells lack.

#### Pericytes

One more cell type in central nervous tissue deserves brief comment. Pericytes are cells involved in the blood-brain barrier. They are found within the basal membrane (BM) of the endothelium, while glial satellites of blood vessels are found outside the endothelial BM. In semithin sections, toluidine blue stains the BM of the endothelium and pericytes can be identified, because of their unique position within the BM (Figure 1G). Pericytes cannot be distinguished from other endothelial cells or glial satellites in thick sections stained with thionin, because the BM is not visible.

### Immunohistochemical Cross-Validation of Cytological Features of GFAP-, Iba-1- and NeuN-Labeled Cells in the Cerebral Cortex

To confirm description of cell types in Nissl stained sections we examined the cytological features of cells in cingulate and lateral prefrontal areas of the monkey cortex in sections processed for specific markers of astrocytes (GFAP), microglia (Iba-1) and neurons (NeuN). Markers were processed with immunoperoxidase technique that stains labeled structures with a brown color. Nonlabeled cells counterstained for Nissl with thionin show the cytological features described above (Figure 7).

Immunohistochemistry for GFAP shows the typical spider like cytoplasm of astrocytes (green arrowheads, Figures 7A–C). Nissl counterstaining confirms that GFAP labeled cells have round and smooth nuclei with a rim of peripheral heterochromatin, some grains of heterochromatin attached to this rim and others in the heterochromatin net, and euchromatin stained light blue. Some astrocytes, significantly neuron satellite astrocytes, are not labeled for GFAP (green arrowhead with asterisk, Figure 7A). Cells showing the features described for neurons, oligodendrocytes, microglia, and endothelial cells are not stained for GFAP (Figures 7A–C).

Iba-1 labeled cells showed ramified processes, which fanned out from the thin perinuclear rim of cytoplasm (Figures 7D,E). The nuclei of these cells were irregular and darkly stained with many small grains of heterochromatin, typical of microglia (Figures 7D,E).

Immunohistochemistry for NeuN labels the cytoplasm and nuclei of most large (black arrows, Figure 7F) and small neurons (orange arrows, Figure 7F) in the cerebral cortex. The Nissl stained nuclei of astrocytes (green arrowheads) and other unlabeled cells stand out among labeled neurons showing the features described above (Figure 7F).

### Ultrastructural Cross-Validation of Cytological Features of Neurons and Glial Cell Types in the Cerebral Cortex

To date, electron microscopy is the most explicit and reliable way to distinguish and unambiguously identify all cell types in the brain. It is the only way to show critical distinguishing features that can be ambiguous or difficult to see under the light microscope, such as the folds of the nuclear envelope, chromatin granules, the nucleolus of glial cells and lipid or protein inclusions. It is also the best way to highlight and understand the effects of multiple factors, including *post-mortem* interval, fixation and processing on tissue and staining quality. We therefore, examined cell types in prefrontal areas of the monkey and human cortex in the electron microscope (Figures 8, 9) to confirm and validate our observations from the optical microscope in Nissl Toluidine Blue- and Thionin-stained histological sections. We also examined area 17 in one case (AM65) with a different fixation to complement the description of oligodendrocytes. A detailed description of fine structural features of neurons and glia in the cortex is beyond the scope of this study, which focuses on features that are visible both in the light and electron microscopes (for more, see Vaughn and Peters, 1971; Cragg, 1976; Miller and Peters, 1981; Ong and Garey, 1991a,b; Peters et al., 1991a,b).

#### Neurons

Neurons have a large cytoplasm, rich in organelles, in particular Nissl bodies (NB), which are packed cisternae of rough endoplasmic reticulum that are labeled in Nissl stained tissue (Figure 8A). Human tissue quality is not optimal, due to poor fixation and long *post-mortem* intervals, and as a result NB and membranes of organelles often break down and the cytoplasm appears granulated (Figures 9A,B). This results in a lighter, more uniform Nissl staining of neuronal cell bodies in human brain tissue compared to the darker, heterogeneous staining in the cytoplasm of neurons from optimally-preserved and well-fixed monkey brain tissue (compare for example Figures 2, 3 with Figures 4, 5). Lipofuscin or other lipid bodies (Lf) in the cytoplasm of neurons are electron dense and appear dark gray or black in the electron microscope (Figure 8A). The nuclear envelope of neurons often has indentations and folds, which are more frequent in large neurons (Figures 8A,B). The nuclei of large neurons appear relatively clear and contain a prominent nucleolus (black arrows, Figures 8A–C). In small neurons the nucleolus (ncl) is smaller and shows perinuclear granules or clumps of heterochromatin (Hchr); some clumps can also be seen under the nuclear envelope or in the central part of the nucleus without contact with perinucleolar heterochromatin (orange arrows, Figures 8D,E). In the nuclei of some neurons there are bundles of filaments (f; Figure 8C). The nucleolus of neurons contains dark, dense regions of ribosome-like granules (pars granulosa, PG) rich in ribonucleoprotein, intertwined with darker regions of condensed fine filaments (pars fibrosa, PF; Figures 8C,E) rich in ribonucleoprotein and DNA. In human neurons the nucleolus appears homogeneous and PF and PG cannot be differentiated (Figures 9A,B).

#### Astrocytes

The cytoplasm of astrocytes (green arrowhead, Figures 8F,G) is thin and irregularly shaped and may contain electron-dense, darkly-stained inclusions. In the case of fibrous astrocytes, which are more abundant in layer I and in the deep layers of the cortex close to the white matter, bundles of fibrils can be visible in the cytoplasm and may correspond to the pinkish threads that are sometimes visible in Nissl stained tissue. The nucleus of astrocytes in the neuropil is typically potato-shaped with a smooth envelope with only occasional indentations, which are more frequent in neuron satellite astrocytes. There is a dark rim of peripheral heterochromatin under the nuclear envelope (Figure 8F). One or two heterochromatin clumps are attached to this rim, but sometimes clumps can also be found in the middle of the nucleus. The euchromatin of astrocytes is darker than in neurons. The nucleolus of astrocytes, when visible, also contains dark PF and PG regions, just like neurons, and is partially surrounded by heterochromatin clumps (Figure 8G).

#### Oligodendrocytes

Oligodendrocytes (silhouette arrowheads, Figures 8H,I, 9B–D), are often seen as satellites to neurons or forming rows, especially in the white matter. The cytoplasm of oligodendrocytes is very thin, irregularly-shaped, and always darkly-stained, likely due to high levels of electron-dense lipids and proteins, including myelin (Figure 8H). However, the cytoplasm of human oligodendrocytes does not preserve well, swells and is usually unstained (Figures 9B,C). The nucleus is round or oval-shaped and very darkly stained in cases fixed with usual protocols (Figure 8H). To appreciate the nuclear distribution of heterochromatin and the nucleolus protocols with more intense fixation are needed, as used in the laboratory of Dr. Alan Peters. In such cases, the nucleus of oligodendrocytes shows multiple darkly-stained heterochromatin clumps that can be positioned near the nuclear envelope or well within the nucleus and around the nucleolus (Figure 8I). Human oligodendrocytes have heterochromatin dispersed in multiple clumps (Figures 9B–D).

#### Microglia

Much like oligodendrocytes, the cytoplasm of microglia, when visible, is very thin, irregularly-shaped, and always darkly-stained (black arrowheads, Figures 8J,K, 9E,F). It may contain inclusion bodies indenting the nucleus (Figure 8J). Microglia nuclei are most often irregular in shape and rarely round, with more granules of heterochromatin compared to oligodendrocytes; these granules of the peripheral heterochromatin and the heterochromatin net are in continuity and form a grid across the nucleus (Figures 8J,K). The nucleolus, when visible, is attached to one central granule (Figures 8J,K). Due to differences in overall health, cause of death and age, there can be regional increases in the density of microglia in human *post-mortem* brain tissue (Figure 9E).

#### Endothelial Cells

The cytoplasm of endothelial cells (orange arrowheads, Figures 8A,L) is thin and shows the typical tight junctions of the blood-brain barrier (Peters et al., 1991b). The nuclei of endothelial cells are flat and show extensive peripheral heterochromatin covering most of the inner surface of the nuclear envelope. Peripheral heterochromatin is in continuity with clumps of heterochromatin net (Figure 8L). The nucleolus is partially surrounded by heterochromatin clumps and is made of PF and PG.

#### Pericytes

In the electron microscope, pericytes are completely encircled by the endothelial BM and their nuclei show homogeneous chromatin (Figure 8L). As already noted in the literature, the nuclei of pericytes are very different compared to endothelial cells and microglia (Peters et al., 1991a,b). The nuclear features of pericytes and their position within the BM of the endothelium are the key features for identifying them. Unfortunately, this can be done only in semithin sections and in the electron microscope. In our thick Nissl-stained sections we could not distinguish pericytes from other endothelial and perivascular cells.

### An Algorithm to Systematically Distinguish Cell Types in the Cerebral Cortex

The key features that allow distinction of neurons and glial cells with Nissl staining at the optical microscope are summarized systematically in Table 2 (monkey, semithin sections) and Table 3 (monkey and human, thick sections). We also highlight these features in hand drawings of selected neurons, astrocytes, oligodendrocytes, microglia and endothelial cells of sections of the monkey and human cortex stained with Nissl (Figure 10).

We used the key features summarized in Tables 2, 3 to develop an algorithm (Figure 11) composed of successive questions to systematically distinguish and identify nerve cell types in the cerebral cortex according to cytological features in toluidine blue and Nissl stained sections. The algorithm classifies cell types in the cerebral cortex into two broad groups. One group includes cells with darkly stained nuclei (microglia and oligodendrocytes; questions 1 and 3) and the other group includes cells with lighter nuclear stain (neurons, astrocytes and endothelial cells; questions 1, 2, 3 and 4). This is an easy distinction to make and the level of stain tends to be correlated with the size of the nucleus. That is, darkly stained nuclei tend to be smaller than lightly stained nuclei. Once a cell is allocated into one of these two broad groups, the algorithm proceeds to distinguish microglia and oligodendrocytes in the darkly stained nucleus group and neurons, astrocytes and endothelial cells in the other group with one question about a key cytological feature: the distribution of heterochromatin in the nucleus.

#### Question 1: Is the Nucleus of the Cell *Darkly Stained*?

If yes, proceed to determine the shape of the darkly stained nucleus; if no, go to question 2.

If the nucleus is elongated, comma shaped, or polylobular, the cell is very likely microglial.

If the nucleus is round or ovoid, the cell is very likely an oligodendrocyte, but some microglial cells may also have round or ovoid nuclei. Go to question 3 to make the distinction between oligodendrocytes and microglia.

#### Question 2: Is the Nucleus of the Cell Surrounded by a *Continuous Rim of Cytoplasm*?

If yes, the cell is very likely a neuron. If no, the cell is very likely an astrocyte or an endothelial cell. Go to question 3 to distinguish between small neurons and astrocytes or endothelial cells.

#### Question 3: What is the Distribution of *Heterochromatin*?

If the nucleus is dark and the peripheral heterochromatin and the heterochromatin net are composed of numerous small granules forming a net or grid, the cell is microglial. The presence of greenish inclusions next to the nucleus (in the cytoplasm) supports this conclusion.

If the nucleus is dark and there are 2–4 rounded granules in the heterochromatin net, the cell is an oligodendrocyte; the presence of perinuclear halo and/or a pinkish crescent of cytoplasm around the nucleus supports this conclusion.

If the nucleus is lightly stained with an “empty” appearance and the only heterochromatin present are small granules around a distinct nucleolus, the cell is a large neuron. This neuron can be pyramidal or non-pyramidal, like fusiform von Economo neurons.

If the nucleus is lightly stained and the nucleolus is partially or totally surrounded by irregular clumps of heterochromatin and there are 1–2 additional granules in the heterochromatin net, the cell is a small neuron. The presence of nuclear folding supports this conclusion and helps distinguish small neurons from astrocytes, especially in humans.

If the cytoplasm is not stained and the nucleus shows a rim of peripheral heterochromatin under the nuclear envelope and several heterochromatin granules are attached to this rim or in the heterochromatin net, then the cell could be an astrocyte or an endothelial cell. Go to question 4 to make the distinction between astrocytes and endothelial cells.

#### Question 4: What is the Staining Status of *Euchromatin*?

If the euchromatin is homogeneously stained, the cell is an astrocyte. A pocket of cytoplasmic yellow granular inclusions next to the nucleus supports this conclusion.

If the staining of the euchromatin is watery, then the cell is endothelial. These cells are usually easy to identify because of the shape of their nuclei wrapping around blood vessels.

### Potential Pitfalls and Ambiguous Profiles

Most cell profiles are easy to distinguish and identify, for instance large neurons. But some cell profiles can be ambiguous and require careful examination of every cytological feature. Here we specifically address the distinction of cell types with ambiguous profiles that may lead to erroneous classifications. The first potential pitfall is the distinction between small neurons and astrocytes, which is reported as one of the most difficult in the stereology literature (von Bartheld et al., 2016). An example of such distinction is in Figure 3B where the vertical orange arrow points to a small neuron and the green arrowhead points to an astrocyte. Both cell profiles have lightly stained nuclei and comparable size, but the rim of cytoplasm around the small neuron is hard to discern. In ambiguous cases, a careful analysis of the heterochromatin distribution will help the decision. In the astrocyte the heterochromatin granules are found in the periphery of the nucleus and in the heterochromatin net as well (green arrowhead, Figure 3B). In contrast, the small neuron shows two thick clumps of heterochromatin in the central part of the nucleus, one of which appears to surround the nucleolus (vertical orange arrow, Figure 3B). Heterochromatin in astrocytes is mostly peripheral, while in small neurons it is mostly central. In Figure 4A there is one small neuron (orange arrow with asterisk) close to four astrocytes (vertical green arrowheads) of comparable size; in this case, the neuron shows nuclear folding, a useful feature to distinguish it from astrocytes.

The second potential pitfall is the distinction of round microglia and oligodendrocytes. Most microglial cells have elongated, polylobular or irregular shape, which facilitates their distinction from oligodendrocytes. However, some microglia have round or ovoid shape (Figure 2A). A careful analysis of heterochromatin is needed for ambiguous darkly stained round nuclei. Microglia have multiple small heterochromatin grains across the nucleus while in oligodendrocytes one or two thicker grains stand out in the nucleus.

We also draw attention to a third potential pitfall that has not been mentioned previously in the literature. Endothelial cells have lightly stained nuclei, unstained cytoplasm and a distribution of heterochromatin comparable to astrocytes. Most endothelial cells are easy to identify because their nuclei mold to the shape of capillaries and larger blood vessels, but in some endothelial cells the molding of the nucleus is not prominent, as seen in Figure 3E. A careful analysis of the euchromatin (question 4, Figure 11) in this ambiguous profile will help identification.

Even with careful examination and experience using the algorithm, disagreements among experimenters unavoidably arise due to two major sources of variability in cell profiles: biological variability in cell morphology, and experimental noise. These sources of variability lead to cell profiles that may be morphological outliers, or abnormally stained, or both. Disagreement among experimenters is discussed further in “On Usefulness and Reliability” Section.

### Algorithm Reliability

We assessed the usefulness of the algorithm by computing inter-experimenter reliability in distinguishing cell types. We required that the raters (MÁG-C, YJJ, BZ, MM, MKJ, IT, JR, and JW) categorize a set of images of Nissl-stained cells. We administered two tests (*N* = 236 and *N* = 114 cells respectively). After the first test, we conducted a second training session in which we discussed pitfalls and ambiguous cells, as we described in “Potential Pitfalls and Ambiguous Profiles” Section. We computed the Krippendorff’s alpha for the aggregated data from both tests, for all raters. Agreement among the raters was high, resulting in a Krippendorff’s alpha value of 0.79 (Hayes and Krippendorff, 2007). When considering only the experienced raters (MÁG-C, YJJ, and BZ), agreement was higher, resulting in an alpha value of 0.92. Data from the group of less experienced or external raters (MM, MKJ, IT, JR, and JW) yielded an alpha value of 0.74. Thus the agreement among experimenters using our algorithm was well above chance (alpha = 0). The average pairwise percent agreement was high (mean ± standard error of mean—82.82 ± 0.01% for the first test and 88.28 ± 0.01% for the second test). These statistical measures indicate that the algorithm enables reliable and reproducible cytological identification. When considering the two tests separately, some improvement in inter-experimenter agreement is suggested, particularly for inexperienced raters. For the first test, Krippendorff’s alpha was 0.75 overall (0.90 for the experienced raters, and 0.70 for the inexperienced raters). For the second test, which followed a session of additional explication of the algorithm, alpha was 0.85 overall (0.93 for the experienced raters, and 0.79 for the inexperienced raters).

As part of the procedure for calculating Krippendorrf’s alpha, we computed a coincidence matrix, consisting of counts of pairwise rater categorizations (Figure 12). Each term on the diagonal is the total number of agreements among pairs of categorizations for the corresponding cell type. The off-diagonal terms are the counts of the disagreements among pairs of raters. The matrix is therefore symmetrical. Perfect agreement among raters (Krippendorff’s alpha = 1) would mean that all off-diagonal terms are zero.

The coincidence matrix (Figure 12) for the complete dataset revealed a high number of inter-experimenter agreements (neurons, *n* = 6614 pairs; astrocytes, *n* = 2866 pairs; oligodendrocytes, *n* = 3432 pairs; microglia, *n* = 1266 pairs; endothelial cells, *n* = 800 pairs) and a smaller number of disagreements. The most common disagreements were astrocyte-endothelial cell (*n* = 341 pairs), neuron-astrocyte (*n* = 327 pairs), oligodendrocyte-microglia (*n* = 267 pairs), and astrocyte-oligodendrocyte (*n* = 175 pairs). These disagreements were anticipated in our discussion of the algorithm. They typically arise due to ambiguity in the Nissl image. We discuss sources of ambiguity and error further in “On Usefulness and Reliability” Section.

## Anticipated Results and Discussion

Precise and systematic descriptions of the cytological features of neuron and glial cell types are needed for unbiased quantification of cells in the brain. We have systematically described the features of neurons, astrocytes, oligodendrocytes, microglial and endothelial cells in sections of the rhesus monkey and human cerebral cortex stained for Nissl at the optical microscope. We have also confirmed distinguishing features of these cells with specific immunohistochemical markers and at the ultrastructural level. The systematic description of key cytological features is summarized in two tables and in an algorithm that categorizes cell types into two groups to simplify cell type identification. One group includes cells with darkly stained nuclei (oligodendrocytes and microglia), and the other group includes cells with lightly stained nuclei (neurons, astrocytes and endothelial cells). We previously used cellular features to estimate neuron and glial cell population in the cerebral cortex (García-Cabezas and Barbas, 2014) and the intercalated masses of the monkey amygdala where neurons and glial cell types could be distinguished with the same criteria used in the cortex (Zikopoulos et al., 2016). We have also used the cellular features that make up the algorithm to estimate the density of neurons in the gray matter of prefrontal cortices of the typical adult human brain and in individuals with autism (Zikopoulos and Barbas, 2010), as well as oligodendrocytes and other glial cells in the white matter below prefrontal cortices in the human brain at the electron microscopic level (Zikopoulos and Barbas, 2010). The formulation of cellular features into an algorithm will help researchers distinguish each cell type reliably and consistently for quantitative unbiased studies in the cerebral cortex.

Our findings and algorithm update and expand Santiago Ramón y Cajal’s observations, who categorized types of cells in the vertebrate nervous system according to the pattern of nuclear chromatin stained with thionin (Ramón Y Cajal, 1896, 1899/2002). At that time many researchers did not differentiate the nucleolus or nucleoli proper from heterochromatin. Levi (1896) and Lenhossék (1897) were the first to distinguish the acidophilic (purple) nucleolus of neurons from the basophilic (blue) perinucleolar heterochromatin, suggesting a different chemical composition and function for each structure. Ramón y Cajal acknowledged this distinction in the second edition of his book on the histology of the nervous system (Ramón Y Cajal, 1909/1952; for a review in English of the cytological features of neurons and astrocytes, see Ramón Y Cajal, 1899/1999, 1909/1995, 1933; also see DeFelipe and Jones, 1988). The cytoplasmic ramifications of oligodendrocytes and microglia do not stain with the colloidal gold technique and Cajal suggested they belong to a “third” element in nervous tissue, where neurons are the first element and astrocytes the second (Ramón Y Cajal, 1913). Using the silver carbonate technique, Pío del Río-Hortega was able to stain the cytoplasm of cells of the “third element” and distinguish them into two cell types: microglia, of mesodermal origin, and oligodendrocytes, of ectodermal origin (Del Río-Hortega, 1919, 1921, 1928; for a review in English of the cytological features of glial cell types see Del Río-Hortega, 1928/2013, 1932; Iglesias-Rozas, 2012).

### Stereological Counts of Nissl-Stained Cortical Sections

Several studies have estimated neuron, astrocyte, oligodendrocyte and microglia numbers in the primate cerebral cortex in Nissl stained sections using unbiased stereological methods (O’Kusky and Colonnier, 1982; Pelvig et al., 2008; Fabricius et al., 2013; Salvesen et al., 2015). Others have estimated neuron and glial cell number without distinction of glial types (Ongur et al., 1998; Rajkowska et al., 1999; Selemon et al., 1999; Cotter et al., 2001; Dombrowski et al., 2001; Lidow and Song, 2001; Christensen et al., 2007). Only one of these studies describes briefly the features of neuron and glial cell types for Nissl stain and confirms their cytological findings with immunohistochemistry (Hou et al., 2012). Most articles contain only brief descriptions and base the distinction of neurons from glial cells on the presence of a distinct nucleolus, which can be misleading because small neurons frequently have small nucleoli surrounded by thick perinucleolar heterochromatin clumps and some large neurons may have two nucleoli.

The glia/neuron ratio for the whole cerebral cortex of the monkey ranges from ~0.57 (Christensen et al., 2007) to 1.2 (Lidow and Song, 2001). In the primary visual cortex of adult monkeys (area 17) the glia/neuron ratio is ~0.49 (O’Kusky and Colonnier, 1982) and in prefrontal areas of rhesus monkeys the glia/neuron ratio ranges between 0.5 and 1.2 (Dombrowski et al., 2001). These figures fall within a close range suggesting a high degree of agreement among observers. Available stereological counts of glia from light microscopy can be compared and cross validated with electron microscopy preparations in the primary visual cortex of monkeys, where O’Kusky and Colonnier (1982) estimated the following proportions: 65% astrocytes, 29% oligodendrocytes, and 6% microglia. A 2D study of glial cell types in area 17 of the monkey using electron microscopy, the gold standard for glial cell type identification, showed 57% astrocytes, 35% oligodendrocytes, and 8% microglia (Peters et al., 1991a) suggesting a high degree of accuracy for optical microscopy studies of glial cell types.

This is the first article to report the degree of inter-observer agreement in the identification of neurons, glial cell types and endothelial cells. Using a novel algorithm in this study our degree of inter-experimenter agreement was high (alpha value was 0.79 for all raters, and 0.92 for experienced raters). The primary sources of disagreement were the distinction between small neurons from astrocytes, oligodendrocytes from microglia, small neurons from oligodendrocytes and astrocytes from endothelial cells. The first two disagreements and the last one are specifically addressed by the algorithm. The distinction between small neurons and astrocytes has been traditionally challenging (reviewed in von Bartheld et al., 2016). This distinction is particularly difficult in the cerebellum, where small granule cells have comparable heterochromatin distribution as astrocytes (Ramón Y Cajal, 1896, 1899/2002; Palay and Chan-Palay, 1974; von Bartheld et al., 2016). Disagreement in distinguishing neurons from oligodendrocytes was not expected, and was restricted to small neurons in cortical layer IV. These potential pitfalls should be taken into account when estimating neuron and glial cell type populations in the cortex.

### On Usefulness and Reliability

In this study, we describe a systematic step-by-step approach to identify cell types in the cerebral cortex with the optical microscope in Nissl stained sections. Use of this approach to quantify the major cell types in the brain can complement and significantly increase the value of studies that use modern molecular or neurochemical labeling techniques to estimate ratios of specific subtypes of cells within a population. Examples of previous studies that used similar approaches include reports showing that inhibitory neurons represent 20–30% of all neurons in the mammalian neocortex and in the frontal cortex of humans they make up approximately 21% of the neuronal population (Hornung and De Tribolet, 1994; Kalus and Senitz, 1996; Benes et al., 2001; Sherwood et al., 2010). These approaches are especially suited for the quantification and comparison of cell types between different cortical areas and brain nuclei. Other approaches, like the isotropic fractionator can be applied to quantify different neuronal and non-neuronal cell types in larger regions of the brain (e.g., cerebellum, cortex), dissected areas, or the entire brain (Herculano-Houzel and Lent, 2005; Azevedo et al., 2009).

Cell identification with the Nissl technique is widely used (O’Kusky and Colonnier, 1982; Dombrowski et al., 2001; Pakkenberg et al., 2003; Christensen et al., 2007; Pelvig et al., 2008; Fabricius et al., 2013), but detailed and systematic descriptions are lacking in the literature. Our algorithm is based on morphological features in Nissl stained sections that can be identified by researchers following brief training. With experience, agreement among researchers increases. However, 100% agreement cannot be expected because various unavoidable sources of noise and variability arise. Some sources of noise and variability are biological; cells of the same type may appear dissimilar and cells of distinct types may appear similar. Cells of a given type fall on a morphological continuum, so ambiguous edge cases arise as a consequence of biological or natural variability. In addition to biological variability, thermal and quantum fluctuations unavoidably arise during experimental procedures involving biochemical reactions including fixation, cryoprotection, cutting and staining. These sources of experimental noise will produce differences in the staining of cells across cases and sections with differential involvement of cell types.

The net result of these two sources of noise and variability, biological and experimental, is ambiguity in the appearance of some stained cells. For example, biological variability is reflected when a microglial cell appears to be round, and therefore difficult to distinguish from an oligodendrocyte. On the other hand, experimental noise is the reason why cells that are typically darkly stained may appear lightly stained and *vice versa*. Thus, there will always be boundary cases: cell profiles that do not contain enough morphological information to make confident categorizations. If cells are morphological outliers, or if staining is noisy, then there may be insufficient information to make accurate and consistent categorizations. In addition to biological and experimental variability in the tissue, researchers may also introduce errors through failure to consistently adhere to the algorithm, or even failure to correctly enter data, while categorizing cells.

A limitation of cell identification through optical microscopy is that there is no staining procedure that can produce ground truth labeling of the various cell types. In other words, the only categorization available for each cell is each researcher’s assessment of its type. For this reason we do not have an independent yardstick with which to assess the accuracy of each researcher. Instead, we use a measure of inter-experimenter agreement: Krippendorff’s alpha. Thus, stereological methods rely on morphological criteria to identify cell types in Nissl stained sections (O’Kusky and Colonnier, 1982; Dombrowski et al., 2001; Pakkenberg et al., 2003; Christensen et al., 2007; Pelvig et al., 2008; Fabricius et al., 2013), but the reliability of these criteria and the inter-observer discrepancies that arise when using them have not been systematically studied.

We have commented on examples of ambiguous cases in the section on potential pitfalls and ambiguous profiles. The ambiguous cases are a minority, and typically involve small neurons vs. astrocytes and microglia vs. oligodendrocytes. Further, the test we performed for assessing the usability of the algorithm by computing inter-experimenter reliability used only a few depths of field, offering less information to the experimenters than a microscope would typically offer.

To further demonstrate the usability of the algorithm and the consistency of results generated by it, we also conducted the cell-labeling test on less experienced experimenters and researchers from other groups. We computed the cumulative Krippendorff’s alpha for all experimenters, and contrasted alphas for experienced vs. less experienced raters. Performance was well above chance even for the inexperienced raters, suggesting that the algorithm can be learned and applied in a consistent manner. Agreement is likely to increase with experience, as picking out subtle differences in morphology requires a trained eye. Both experienced and less experienced experimenters showed strong agreement in identifying neurons. Glial and endothelial cells were harder to distinguish, particularly for inexperienced raters. Nevertheless, given the reliability of neuron identification, glia-neuron ratios are likely to be consistent even among inexperienced raters, provided the stereological procedures are based on an explicit classification procedure such as the one described here. As such, the approach described here, including the protocol and the inter-experimenter analysis of correspondence, can be used to train researchers in order to reduce variability and ensure reproducibility and reliability of analyses. Assessed metrics can be used to determine when studies can be undertaken, which optimally would be when raters reach a level of agreement within the “expert” range (alpha > 0.90).

### Implications for the Study of Cortical Structure and Its Disruption in Neurological and Psychiatric Disorders

Accurate distinction of neurons and glial cell types is fundamental for understanding systematic variation of each cell type number and density as well as glia to neuron ratio across cortical areas (Dombrowski et al., 2001; García-Cabezas and Barbas, 2014; Barbas, 2015; Barbas and García-Cabezas, 2016). Moreover, such studies in neurotypical human and non-human primate brains are needed to establish a consistent framework that can be used to compare cell numbers, densities and ratios in disease. Examples include probing the differential involvement of loss of neurons and glia in aging (Pakkenberg et al., 2003; Pelvig et al., 2008; Fabricius et al., 2013), neurodegeneration (Pelvig et al., 2003), and psychiatric disorders (Thune et al., 2001; Stark et al., 2004). Distinction of astrocytes, oligodendrocytes and microglia is relevant because each glial type plays different roles in brain function and disease. For instance, reduction of astrocytes has been implicated in depression (Ongur et al., 1998; Rajkowska et al., 1999; Gittins and Harrison, 2011; Torres-Platas et al., 2011; reviewed in Rajkowska and Stockmeier, 2013). In psychiatric disorders, neurons and glial cells may not show structural changes detectable with routine stains but careful unbiased stereological counts may demonstrate increase or decrease in number, density or ratios of particular cell types and subtypes (Preuss and Kaas, 1996; Simms et al., 2009). Accurate identification of neurons and glia is also important for the study of neurodevelopmental disorders like autism, where research suggests that cortical dysplasia and disorganization of neurons and glia in childhood (Wegiel et al., 2010; Morgan et al., 2012; Casanova et al., 2013; Stoner et al., 2014) may not persist in adulthood, at least in some prefrontal cortices (Zikopoulos and Barbas, 2010, 2013). The algorithm presented here when used for unbiased stereological estimates of astrocyte, microglia and oligodendrocyte number and density and of neuron to glial cell type ratio across cortical areas in the human brain will provide a basis to study possible differential involvement of cortical areas in brain disorders.

## Author Contributions

MÁG-C and BZ designed and performed the experiments, collected and analyzed the data, and wrote the initial drafts of the manuscript. HB and YJJ assisted with the design of the study and performed analyses. All authors contributed to writing the manuscript and approved its final version.

## Funding

This work was supported by NIH grants from the National Institute of Neurological Disorders and Stroke (R01NS024760); the National Institute of Mental Health (R01MH057414 and R01MH101209); and by Center of Excellence for Learning in Education, Science and Technology, a National Science Foundation Science of Learning Center (grant number NSF SBE-0354378). MÁG-C is the recipient of a 2014 National Alliance for Research on Schizophrenia and Depression (NARSAD) Young Investigator Grant from the Brain and Behavior Research Foundation (grant number 22777, P&S Fund Investigator).

## Conflict of Interest Statement

The authors declare that the research was conducted in the absence of any commercial or financial relationships that could be construed as a potential conflict of interest.

## Footnotes

^1^http://sites.bu.edu/brainlab/neurocytology-test/
